# Systemic Inflammatory Response During Coronary Artery Bypass Grafting with Cardiopulmonary Bypass: Cellular and Molecular Mechanisms

**DOI:** 10.3390/cells15181717

**Published:** 2026-09-21

**Authors:** Dejan M. Lazović, Dragan Cvetković, Milica Karadžić Kočica, Dragan Ivanišević, Vojkan Aleksić, Mladen J. Kočica, Danko Grujić, Selena Nešić, Jovana Klać, Milica Grujić, Sashko Nikolov, Stefan Juričić

**Affiliations:** 1 Clinic for Cardiac Surgery, University Clinical Center of Serbia, 11000 Belgrade, Serbiadanko0702@gmail.com (D.G.); 2 Faculty of Medicine, University of Belgrade, 11000 Belgrade, Serbia; 3 Center for Anesthesiology, Reanimatology and Intensive Care Medicine, University Clinical Center of Serbia, 11000 Belgrade, Serbia; 4 Clinic for Cardiology, University Clinical Center of Serbia, 11000 Belgrade, Serbia; 5 Institute of Rheumatology, 11000 Belgrade, Serbia; 6 Faculty of Medical Sciences, Goce Delcev University, 2000 Stip, North Macedonia; dr.nikolovsasko@gmail.com

**Keywords:** cardiopulmonary bypass, coronary artery bypass grafting, systemic inflammatory response syndrome (SIRS), complement activation, cytokine storm, neutrophil extracellular traps (NETs), endothelial dysfunction, ischemia–reperfusion injury, Toll-like receptors, hemoadsorption

## Abstract

Coronary artery bypass grafting (CABG) utilizing cardiopulmonary bypass (CPB) remains a cornerstone of treatment for advanced multivessel coronary artery disease. During the ischemic period, myocardial cells become injured and increasingly depend on anaerobic metabolism. After reperfusion, exposure of blood components to artificial surfaces, combined with ischemia–reperfusion injury, surgical trauma, and gut translocation of endotoxins, triggers a complex systemic inflammatory response syndrome (SIRS). This inflammatory cascade involves a tightly regulated network of humoral cascades (complement, contact, coagulation, and fibrinolytic systems) and cellular effectors (neutrophils, monocytes, endothelial cells, and platelets). Molecular signaling pathways, notably Toll-like receptor 4 (TLR4) activation, nuclear factor kappa B (NF-κB) nuclear translocation, mitogen-activated protein kinase (MAPK) phosphorylation, and NLRP3 inflammasome assembly, drive a cytokine storm characterized by massive releases of interleukin-6 (IL-6), tumor necrosis factor-alpha (TNF-α), interleukin-1 beta (IL-1β), and interleukin-8 (IL-8). These molecular events lead to endothelial barrier disruption, vasoplegic shock, acute lung injury, myocardial dysfunction, and acute kidney injury. This comprehensive narrative review provides a detailed synthesis of the cellular and molecular mechanisms underlying CPB-induced SIRS, highlights recent advances in neutrophil extracellular trap (NET) dynamics and microvascular injury, and evaluates contemporary pharmacological and bioengineering therapeutic strategies designed to mitigate postoperative organ dysfunction.

## 1. Introduction

Coronary artery bypass grafting (CABG) is one of the most frequently performed cardiac surgical procedures worldwide [1]. While on-pump CABG using cardiopulmonary bypass (CPB) provides a motionless, bloodless surgical field and maintains systemic perfusion, it exposes the patient to significant non-physiological stress [2]. The contact of circulating blood with the synthetic materials of the extracorporeal circuit, combined with surgical trauma, aortic cross-clamping, hypothermia, cardioplegic arrest, and ischemia–reperfusion (I/R) injury, triggers a pronounced systemic inflammatory response syndrome (SIRS) [3,4].

Although mild inflammatory activation is a necessary homeostatic response to surgical trauma, an exaggerated or dysregulated inflammatory response occurs in up to 30% of patients undergoing CPB [5,6]. Clinically, CPB-induced SIRS manifests across a spectrum ranging from benign, transient fever and fluid retention to severe vasoplegic shock, acute respiratory distress syndrome (ARDS), acute kidney injury (AKI), cognitive dysfunction, and multiple organ dysfunction syndrome (MODS) [7,8].

Over the past three decades, significant technological refinements—such as biocompatible circuit coatings, miniaturized CPB circuits, modified ultrafiltration, and off-pump CABG (OPCAB)—have reduced surgical morbidity [9,10]. Nevertheless, CPB-induced SIRS remains a primary driver of postoperative mortality, prolonged intensive care unit (ICU) length of stay, and elevated healthcare costs [11,12]. Understanding the intimate cellular and molecular networks that drive this inflammatory phenomenon is imperative for identifying novel biomarker targets and tailoring targeted therapeutic interventions.

### Methods

**Figure 1:**
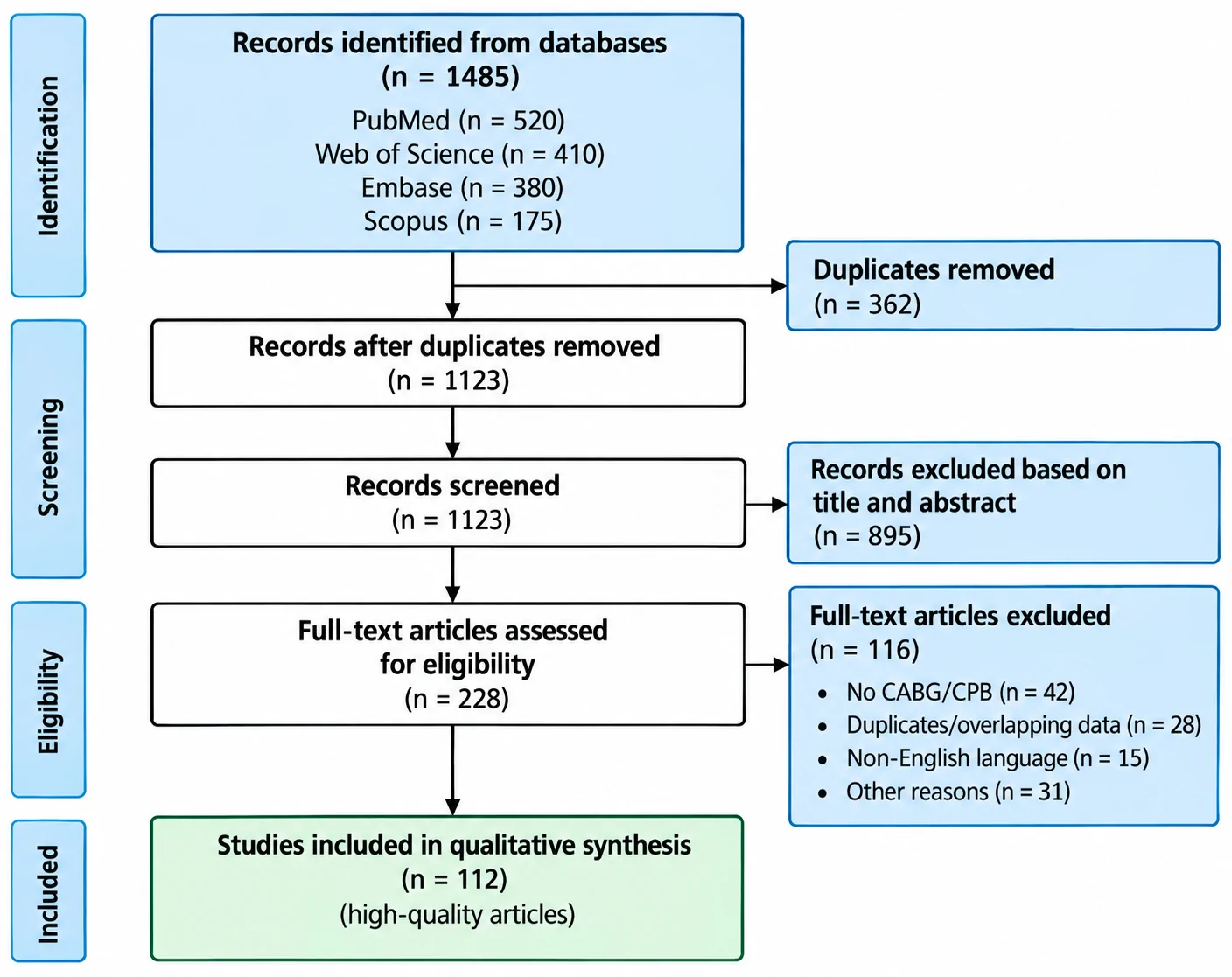
PRISMA-style flow diagram of the literature search and study selection process. The literature search of PubMed/MEDLINE, Web of Science, Embase, and Scopus identified 1485 records published between January 2000 and July 2026. After removal of 362 duplicate records, 1123 unique records underwent title and abstract screening, resulting in the exclusion of 895 records. Full texts of 228 articles were subsequently assessed for eligibility. Of these, 116 articles were excluded because they did not provide relevant CABG/CPB data or specific molecular endpoints, contained duplicate or overlapping datasets, were published in a non-English language, or had other methodological or publication-related limitations. Finally, 112 high-quality articles met the predefined eligibility criteria and were included in the qualitative narrative synthesis.

This review provides a narrative synthesis of the cellular, molecular, and pathophysiological mechanisms underlying the systemic inflammatory response during CABG with CPB, detailing humoral cascade activation, leukocyte effector mechanisms, intracellular signaling cascades, tissue-specific organ injury, and current therapeutic strategies. We conducted a comprehensive electronic search across PubMed/MEDLINE, Embase, Web of Science, and Scopus for articles published between January 2000 and July 2026. The search strategy combined Medical Subject Headings (MeSH) terms and high-impact keywords using Boolean logic (AND, OR). In addition to automated primary database queries, we manually cross-referenced reference lists from high-impact review articles and seminal experimental studies to identify the relevant secondary literature. Studies were evaluated based on predefined inclusion and exclusion criteria designed to prioritize molecular, cellular, and pathophysiological evidence (Figure 1).

Inclusion Criteria:
  1. Peer-reviewed original research (in vitro, animal models, clinical trials, and prospective observational studies) detailing specific molecular or cellular pathways of CPB-induced inflammation during CABG.
  2. Articles analyzing primary inflammatory cascades (e.g., complement system activation, contact system activation, leukocyte–endothelial interactions, platelet activation, oxidative stress, and cytokine/chemokine dynamics).
  3. Articles published in English in peer-reviewed journals between January 2000 and July 2026.
Exclusion Criteria:
  1. Studies evaluating off-pump CABG (OPCAB) exclusively without a CPB comparative arm or insights into CPB-induced mechanisms.
  2. Purely clinical or epidemiological outcome studies lacking cellular, molecular, or biochemical mechanistic data.
  3. Case reports, editorials, letters to the editor, conference abstracts, non-peer-reviewed preprints, and non-English publications.
  4. Studies with insufficient methodological reporting or duplicate datasets.
A multi-stage screening strategy was performed to select the final literature cohort:
  1. Identification: The initial database search yielded 1485 citations (PubMed: 520, Web of Science: 410, Embase: 380, Scopus: 175).
  2. Deduplication: A total of 362 duplicate entries were identified and removed, leaving 1123 unique records.
  3. Screening: Title and abstract screening led to the exclusion of 895 records that failed to meet the foundational criteria.
  4. Eligibility Evaluation: Full texts of the remaining 228 articles were comprehensively reviewed against the inclusion/exclusion criteria. A total of 116 articles were excluded due to lack of specific molecular endpoints (*n* = 42), exclusive OPCAB focus (*n* = 31), methodological limitations (*n* = 28), or unsuitable publication format/language (*n* = 15).
  5. Included Studies: A final total of 112 high-quality articles were selected and included in the qualitative narrative synthesis.

## 2. Etiology and Triggers of CPB-Induced Inflammatory Activation

The pathogenesis of SIRS during CABG with CPB is multifactorial, broadly categorized into material-dependent (contact activation) and material-independent mechanisms [13,14].

### 2.1. Material-Dependent Triggers

The primary trigger of CPB-induced inflammation is the continuous exposure of patient blood to the expansive surface area (~1.5–2.5 m^2^) of synthetic polymers present in the tubing, oxygenator, arterial filter, and reservoir [15,16]. These artificial surfaces lack the endogenous endothelial surface layer, including the glycocalyx and intrinsic anticoagulants such as thrombomodulin and heparan sulfate [17]. Rapid adsorption of plasma proteins—predominantly fibrinogen, fibronectin, immunoglobulin G (IgG), and factor XII (Hageman factor)—onto the foreign surface within seconds of circuit priming initiates autoactivation of factor XII and alternative/classical complement pathways [18,19].

Furthermore, mechanical forces generated by roller or centrifugal pumps cause shear stress, cavitation, and turbulence, inducing direct mechanical damage to circulating erythrocytes, platelets, and leukocytes [20,21]. The liberation of cell-free hemoglobin and microparticles acts as potent pro-inflammatory damage-associated molecular patterns (DAMPs) [22].

### 2.2. Material-Independent Triggers

Several non-surface mechanisms amplify inflammatory pathways independently of synthetic circuit interaction:

1. Surgical Trauma: Direct tissue incision, sternotomy, pericardial disruption, and surgical retraction release intracellular contents, including high-mobility group box 1 (HMGB1), heat shock proteins (HSPs), and mitochondrial DNA (mtDNA), into the circulation [23,24].
2. Ischemia–Reperfusion (I/R) Injury: Aortic cross-clamping induces global myocardial ischemia, while lung ventilation arrest causes pulmonary ischemia [25]. Subsequent cross-clamp removal and reperfusion trigger massive bursts of reactive oxygen species (ROS), calcium overload, and mitochondrial permeability transition pore (mPTP) opening [26,27].
3. Endotoxemia and Gut Barrier Dysfunction: Splanchnic hypoperfusion during hypothermic CPB, non-pulsatile blood flow, and systemic vasodilation lead to intestinal mucosal ischemia [28]. Impairment of the intestinal epithelial barrier permits translocation of lipopolysaccharide (LPS/endotoxin) from Gram-negative gut flora into the portal and systemic circulation, driving Toll-like receptor 4 (TLR4) activation [29,30].
4. Thermal Changes: Hypothermia alters membrane fluidity, retards enzymatic inactivation of pro-inflammatory mediators, and impairs platelet function. Rapid rewarming then activates endothelial cells and stimulates cytokine production [31,32].
5. Heparin–Protamine Complexes: Neutralization of systemic heparin with protamine sulfate at the conclusion of CPB forms insoluble macromolecular complexes that activate the classical complement pathway and induce pulmonary vasoconstriction and systemic hypotension [33,34].

## 3. Humoral Cascade Systems

The activation of four interrelated plasma protein cascades—the complement, contact-kinin, coagulation, and fibrinolytic systems—forms the early humoral axis of CPB-induced SIRS [35].

### 3.1. The Complement System

Complement activation is a cornerstone of CPB pathophysiology and occurs via three distinct pathways: classical, alternative, and lectin [36,37].

- Alternative Pathway: Predominantly activated during the initial minutes of CPB via spontaneous hydrolysis of C3 (C3(H2O)) upon contact with foreign surfaces, forming the alternative pathway C3 convertase (C3bBb) [38].
- Classical Pathway: Activated primarily post-CPB by heparin–protamine immune complexes, anti-protamine antibodies, and naturally occurring antibodies binding to damaged vascular surfaces [39].
- Lectin Pathway: Initiated by mannose-binding lectin (MBL) and ficolins binding to stress-induced patterns on ischemic endothelial cells [40].

Cleavage of C3 and C5 generates potent anaphylatoxins: C3a and C5a. C5a binds to its high-affinity G-protein coupled receptors (C5aR1/CD88 and C5aR2) on neutrophils and monocytes, driving chemoattraction, degranulation, ROS production, and upregulated expression of β_2_-integrins(CD11b/CD18) [41,42]. The terminal sequence generates the membrane attack complex (C5b-9, or MAC), which inserts into cell membranes, inducing sublytic endothelial cell activation, pro-inflammatory cytokine release, and cell lysis [43,44].

### 3.2. Contact-Kinin and Coagulation Cascades

Adsorption of Factor XII (FXII) to artificial surfaces induces a conformational change, converting it to FXIIa [45]. FXIIa cleaves plasma prekallikrein to kallikrein, which feeds back to amplify FXII activation and cleave high-molecular-weight kininogen (HMWK) to liberate bradykinin [46]. Bradykinin is a potent mediator of smooth muscle relaxation and vascular hyperpermeability via B_2 receptors, significantly contributing to CPB-associated hypotension and tissue edema [47].

Concurrently, FXIIa initiates the intrinsic coagulation pathway by activating Factor XI [48]. Simultaneously, surgical trauma and tissue injury expose extrinsic Tissue Factor (TF), which complexes with Factor VIIa [49]. This dual pathway activation leads to massive generation of thrombin. Beyond converting fibrinogen to fibrin, thrombin acts through Protease-Activated Receptors (PAR-1 and PAR-4) on endothelial cells and platelets, inducing P-selectin translocation, IL-6 and IL-8 release, and microvascular thrombosis [50,51].

### 3.3. Fibrinolysis

Systemic heparinization during CPB does not fully suppress microvascular fibrin deposition. The release of tissue plasminogen activator (t-PA) from ischemic endothelial cells converts plasminogen to plasmin [52]. Plasmin degrades fibrin clots, degrades platelet surface GPIb receptors, activates Factor XII, and directly stimulates complement cascades, perpetuating both bleeding coagulopathies and inflammatory loops [53].

## 4. Cellular Mediators and Effector Dynamics

Humoral mediators rapidly mobilize circulating and tissue-resident inflammatory cells, initiating intercellular cross-talk (Table 1) [54].

### 4.1. Neutrophils and NETosis

Polymorphonuclear neutrophils (PMNs) are the primary cellular responders during CPB [55]. C5a, TNF-α, and platelet-activating factor (PAF) induce rapid neutrophil margination, pulmonary sequestration, and extravasation [56]. Activated neutrophils release cytotoxic granules containing myeloperoxidase (MPO), neutrophil elastase (NE), matrix metalloproteinases (MMP-8, MMP-9), and gelatinase, causing degradation of extracellular matrix components and basement membranes [57,58].

A critical discovery in CPB pathophysiology is the induction of Neutrophil Extracellular Traps (NETs)—a process termed NETosis [59,60]. Triggered by shear stress, ROS, C5a, and activated platelets, neutrophils extrude decondensed chromatin fibers decorated with histones, MPO, and elastase into the circulation [61]. While evolutionarily designed to capture pathogens, intravascular NETs act as double-edged swords: they induce endothelial toxicity, promote microvascular thrombosis, activate the intrinsic coagulation pathway, and cause organ failure, particularly in the lungs and kidneys [62,63].

### 4.2. Monocytes and Macrophages

**Figure 2:**
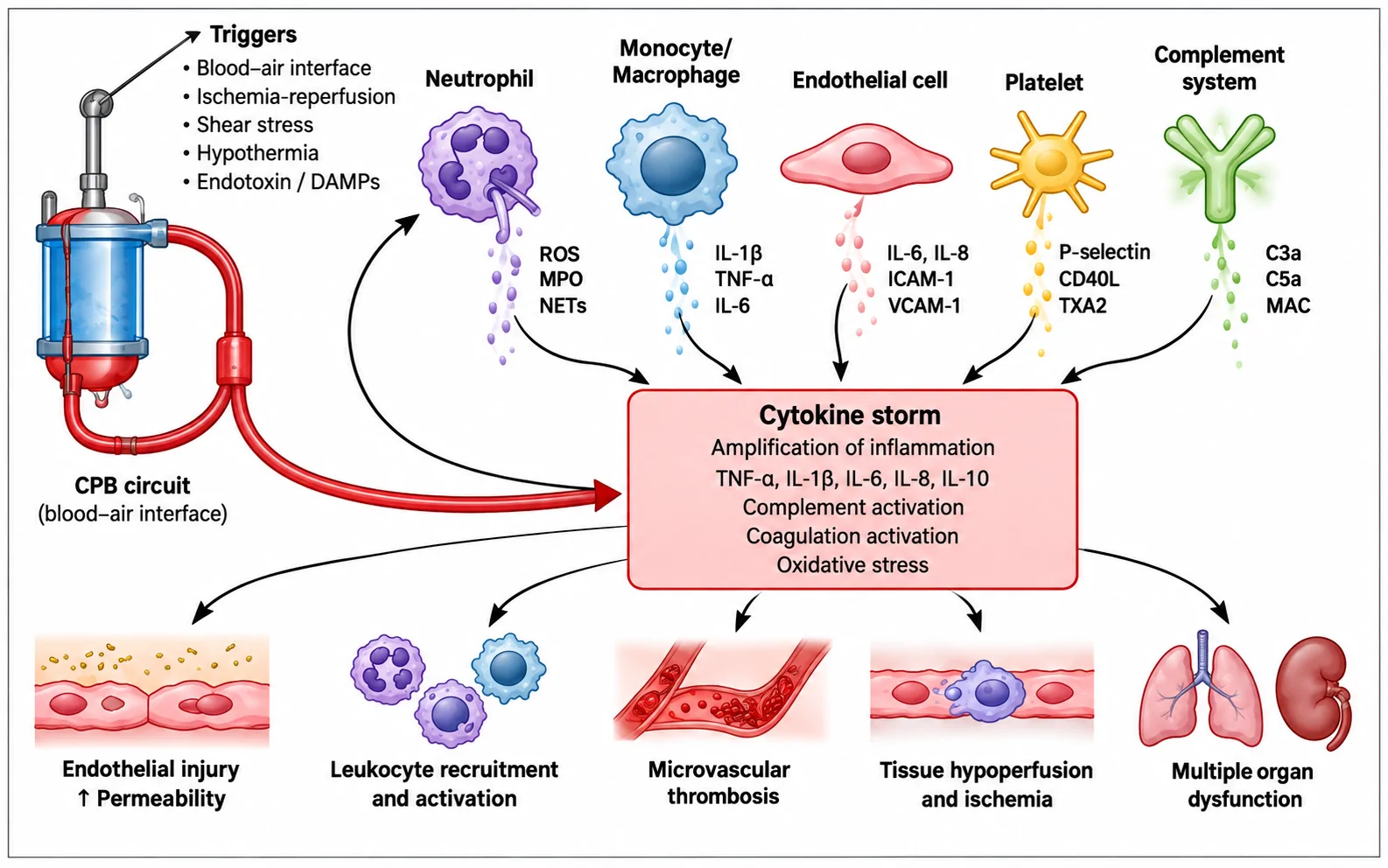
Cellular interactions and amplification of the systemic inflammatory response during cardiopulmonary bypass (CPB). Blood–air contact, ischemia–reperfusion, shear stress, and other CPB-related stimuli activate neutrophils, monocytes/macrophages, endothelial cells, platelets, and the complement system, leading to cytokine release, endothelial injury, microvascular dysfunction, and systemic inflammation. ↑ indicates increased or upregulated activity.

Circulating monocytes undergo profound phenotypic and functional alterations during CPB (Figure 2). Contact with synthetic materials and endotoxins converts classic CD14^^{++}^CD16^^^-monocytes into intermediate (CD14^^{++}^CD16^^+^) and non-classic (CD14^^^+CD16^^{++}^) pro-inflammatory subsets [64]. Monocytes upregulate tissue factor (TF) expression and secrete peak levels of pro-inflammatory cytokines—including IL-6, TNF-α, IL-1β, and IL-8—reaching maximum serum concentrations 2 to 6 h post-reperfusion [65,66]. Furthermore, alveolar and tissue-resident macrophages become hyper-responsive, sustaining inflammatory signals well into the postoperative period [67].

### 4.3. Endothelial Cell Activation and Glycocalyx Shedding

The vascular endothelium transitions from a quiescent, anti-thrombotic state to an activated, pro-adhesive, pro-coagulant phenotype [68]. Endothelial cell activation occurs in two distinct phases:

- Type I Activation (Rapid/Transient): Stimulated by histamine, bradykinin, and thrombin; characterized by exocytosis of Weibel–Palade bodies releasing P-selectin and von Willebrand factor (vWF) within minutes [69].
- Type II Activation (Sustained): Driven by TNF-α, IL-1β, and lipopolysaccharide (LPS); involves de novo gene transcription of adhesion molecules, including E-selectin, Intercellular Adhesion Molecule-1 (ICAM-1), and Vascular Cell Adhesion Molecule-1 (VCAM-1) over hours [70,71].

Concurrently, shear stress, ischemia–reperfusion, and circulating proteases induce shedding of the endothelial glycocalyx—a carbohydrate-rich meshwork (0.5–3 μm thick) lining the luminal surface composed of heparan sulfate, syndecan-1, and hyaluronan [72,73]. Glycocalyx degradation impairs microvascular autoregulation, increases capillary permeability, accelerates leukocyte adhesion, and leads to tissue edema [74].

### 4.4. Platelets and Leukocyte–Platelet Aggregates

Platelets undergo activation via mechanical shear stress, contact with circuit polymers, and agonist exposure (adenosine diphosphate [ADP], thrombin, epinephrine) [75]. Activated platelets express P-selectin (CD62P), which binds to P-selectin glycoprotein ligand-1 (PSGL-1) on neutrophils and monocytes [76]. This forms circulating platelet–leukocyte aggregates (PLAs), which represent sensitive markers of systemic inflammation [77]. PLAs exhibit mutual activation: platelets enhance neutrophil oxidative burst and NETosis, while activated leukocytes stimulate local fibrin deposition and microvascular occlusion [78].

## 5. Intracellular Signaling Cascades and Transcriptional Regulation

**Figure 3:**
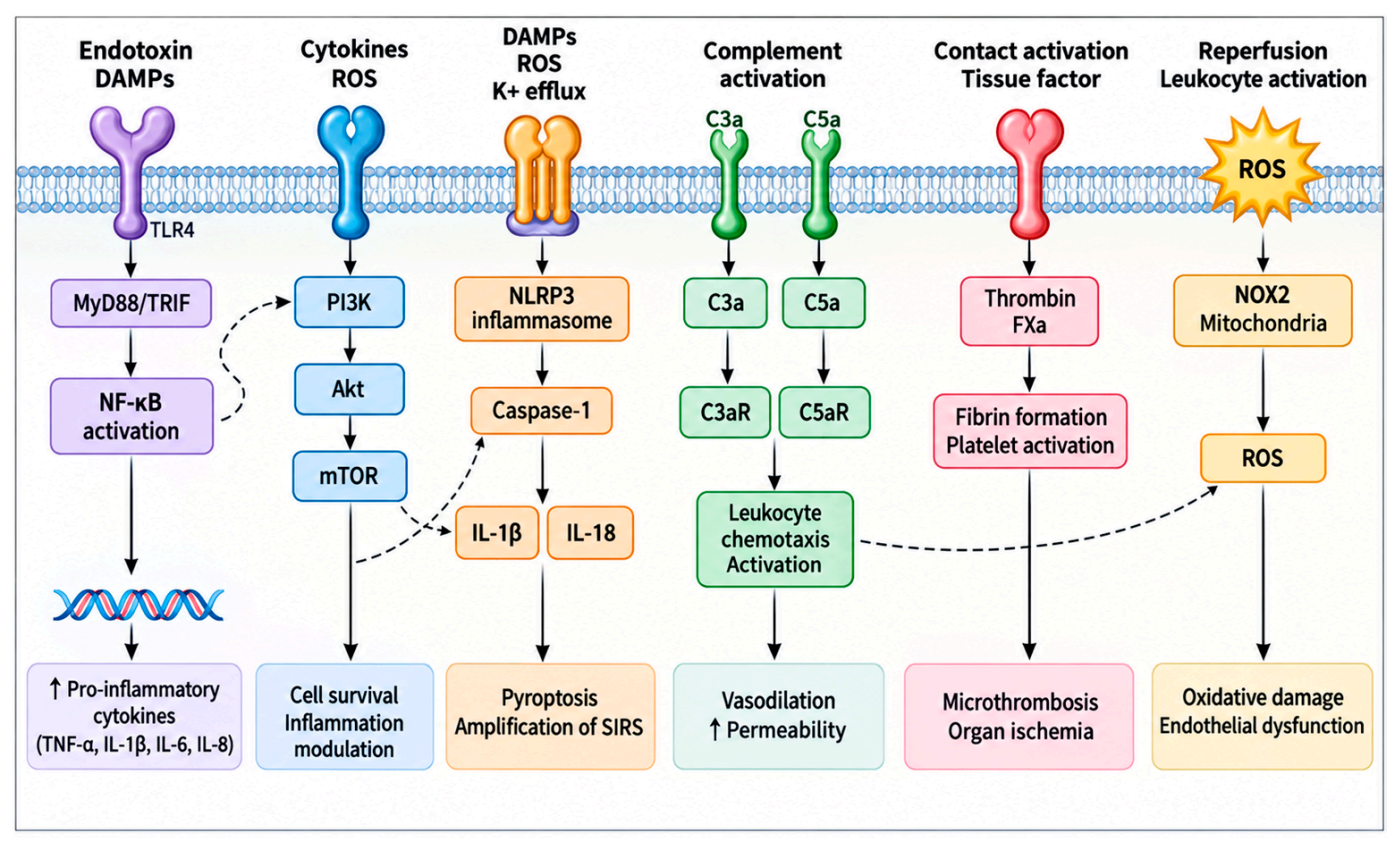
Major molecular signaling pathways involved in the systemic inflammatory response during CPB. Toll-like receptor, NF-κB, NLRP3 inflammasome, complement, coagulation, oxidative stress, PI3K/Akt/mTOR, and MAPK pathways interact to amplify inflammation, endothelial dysfunction, microvascular injury, and subsequent organ dysfunction. ↑ indicates increased or upregulated activity.

Cardiopulmonary bypass (CPB) initiates a complex network of intracellular signaling pathways that integrate mechanical, inflammatory, oxidative, metabolic, and ischemia–reperfusion stimuli. Exposure of blood to the artificial surfaces of the extracorporeal circuit, surgical trauma, altered shear stress, hypothermia, ischemia–reperfusion, endotoxemia, and cellular injury generate multiple danger signals. These signals are recognized by pattern-recognition receptors (PRRs), including Toll-like receptors (TLRs), NOD-like receptors (NLRs), and receptors for advanced glycation end products (RAGE). Their activation converges on several interconnected signaling pathways, particularly NF-κB, MAPK, PI3K/Akt/mTOR, JAK/STAT, TGF-β/SMAD, NLRP3 inflammasome, and Nrf2-dependent pathways. CPB-induced signaling also involves NADPH oxidases, mitochondrial reactive oxygen species (ROS), intracellular calcium signaling, endothelial nitric oxide pathways, and leukocyte adhesion signaling. Importantly, these pathways exhibit extensive cross-talk, creating amplification loops that can sustain systemic inflammation after separation from CPB (Figure 3) [79].

### 5.1. Toll-like Receptors (TLRs) and Pattern Recognition

Toll-like receptor 4 (TLR4) represents one of the major pattern-recognition receptors involved in inflammatory signaling during CPB and ischemia–reperfusion injury [80]. Although TLR4 is classically recognized as a receptor for bacterial lipopolysaccharide, it can also recognize endogenous damage-associated molecular patterns (DAMPs), including HMGB1, heat-shock proteins, and other molecules released during tissue injury. CPB-associated tissue trauma, ischemia–reperfusion, oxidative stress, and intestinal barrier dysfunction can therefore provide multiple stimuli capable of activating TLR4. After ligand recognition, TLR4 recruits adaptor proteins, including MyD88 and TIRAP, activating IRAK4, IRAK1, TRAF6, and TAK1. TAK1 subsequently activates the IKK complex and several MAPK pathways. IKK-mediated phosphorylation and degradation of IκBα releases NF-κB, particularly the p65/p50 heterodimer, allowing its translocation into the nucleus. Nuclear NF-κB binds κB regulatory elements and promotes transcription of TNF-α, IL-1β, IL-6, IL-8, chemokines, adhesion molecules, and other inflammatory mediators [81,82]. TLR4 can also activate a MyD88-independent TRIF/TRAM pathway, resulting in IRF3 activation and type I interferon production, while contributing to a later phase of NF-κB activation. In CPB, this pathway provides an important molecular connection between tissue injury and systemic innate immune activation. TLR4 signaling may therefore amplify leukocyte activation, endothelial dysfunction, cytokine production, and postoperative organ injury. The TLR4 pathway also interacts with ROS, MAPK, PI3K/Akt, and inflammasome signaling, making it a central upstream component of the CPB-induced inflammatory network [83].

### 5.2. Nuclear Factor-Kappa B (NF-κB), MAPK and TGF-β/SMAD Signaling Pathway

NF-κB is a central transcriptional regulator of the inflammatory response associated with CPB [84]. Under resting conditions, NF-κB is retained in the cytoplasm through association with inhibitory IκB proteins. CPB-associated stimuli, including TLR activation, cytokines, oxidative stress, ischemia–reperfusion, and DAMPs, activate the IKK complex. IKKβ phosphorylates IκBα, leading to its ubiquitination and proteasomal degradation. The released NF-κB p65/p50 complex then translocates into the nucleus and binds κB-responsive DNA elements. This promotes transcription of genes encoding TNF-α, IL-1β, IL-6, IL-8, adhesion molecules, cyclooxygenase-2, inducible nitric oxide synthase, and other inflammatory mediators [85]. NF-κB activation therefore links upstream recognition of tissue injury to the amplification and propagation of systemic inflammation. Experimental and clinical evidence indicates that NF-κB activation is associated with ischemia–reperfusion and inflammatory responses during cardiac surgery. NF-κB also interacts with MAPK/AP-1 signaling, creating synergistic transcriptional responses. In endothelial cells, NF-κB activation promotes expression of ICAM-1, VCAM-1, and selectins, thereby facilitating leukocyte adhesion and transmigration. In circulating leukocytes, sustained NF-κB activation enhances cytokine production and cellular activation [86]. Importantly, NF-κB is not exclusively detrimental, because its biological effects depend on timing, cellular context, and the balance between pro- and anti-inflammatory signaling. Nevertheless, excessive or prolonged NF-κB activation during and after CPB is considered an important molecular mechanism contributing to systemic inflammatory response and postoperative organ dysfunction [87,88].

Parallel to NF-κB, Mitogen-Activated Protein Kinase (MAPK) pathways—comprising p38 MAPK, extracellular signal-regulated kinase (ERK1/2), and c-Jun N-terminal kinase (JNK)—are activated by ischemia–reperfusion ROS and physical shear stress [89,90]. MAPKs include three principal branches relevant to inflammation: extracellular signal-regulated kinases 1/2 (ERK1/2), c-Jun N-terminal kinases (JNK), and p38 MAPK. TLR4 activation through MyD88, TRAF6, and TAK1 provides an important upstream mechanism for activation of these pathways. ERK1/2 signaling is particularly involved in cellular activation, proliferation, and transcriptional regulation, whereas JNK and p38 are strongly associated with cellular stress and inflammatory responses. Activated MAPKs phosphorylate downstream transcription factors, including AP-1 components such as c-Jun and c-Fos. AP-1 cooperates with NF-κB to enhance transcription of inflammatory cytokines, chemokines, adhesion molecules, and enzymes involved in oxidative and inflammatory responses. CPB-associated alterations in leukocyte signaling have been directly linked to changes in p38 MAPK, Akt, and PLCγ2 phosphorylation, demonstrating that CPB modifies intracellular signaling involved in leukocyte recruitment. MAPK activation can also increase NADPH oxidase activity, thereby enhancing ROS generation. ROS, in turn, can further activate MAPK signaling, creating a positive feedback loop between oxidative stress and inflammation. Excessive JNK and p38 activation may promote mitochondrial dysfunction, apoptosis, and endothelial injury during ischemia–reperfusion. Consequently, MAPK signaling serves as an important molecular bridge linking mechanical and inflammatory stimuli during CPB to leukocyte activation, cytokine production, oxidative stress, and cellular injury [91].

Transforming growth factor-β (TGF-β) signaling represents a regulatory pathway involved in inflammation, endothelial biology, extracellular matrix remodeling, and tissue repair [92]. TGF-β ligands bind type II receptors, which recruit and phosphorylate type I serine/threonine kinase receptors. Activated type I receptors phosphorylate receptor-regulated SMAD proteins, particularly SMAD2 and SMAD3. These proteins associate with SMAD4 and translocate into the nucleus to regulate gene transcription [93]. During and after CPB, tissue injury, platelet activation, endothelial perturbation, and inflammatory cytokines may influence TGF-β signaling. Depending on timing and cellular context, the pathway can contribute to both resolution and persistence of inflammation. TGF-β signaling influences endothelial and fibroblast phenotypes and may participate in extracellular matrix remodeling after myocardial and vascular injury. It also interacts with inflammatory pathways such as NF-κB and MAPK. In prolonged inflammatory states, excessive TGF-β signaling may promote fibrosis and maladaptive remodeling [94]. Conversely, controlled TGF-β activity may participate in resolution and tissue repair. Therefore, TGF-β/SMAD signaling should be interpreted as a context-dependent regulatory pathway rather than exclusively pro- or anti-inflammatory [95]. In CPB, its major importance may become more apparent during the transition from acute inflammatory injury to repair and remodeling (Table 2).

### 5.3. NLRP3 Inflammasome Activation

The NLRP3 inflammasome has emerged as an important component of innate immune activation during ischemia–reperfusion and cardiac surgery [96]. NLRP3 functions as an intracellular sensor that responds to multiple forms of cellular stress rather than a single specific ligand. During CPB, potential activating signals include mitochondrial ROS, extracellular ATP, potassium efflux, calcium dysregulation, DAMPs, tissue injury, and ischemia–reperfusion. NLRP3 activation promotes assembly of a multiprotein complex containing NLRP3, the adaptor ASC, and pro-caspase-1. Caspase-1 activation subsequently promotes cleavage of pro-IL-1β and pro-IL-18 into their biologically active forms. Caspase-1 can also induce pyroptosis through cleavage of gasdermin D, resulting in inflammatory cell death and release of intracellular inflammatory mediators. NLRP3 activation can therefore amplify inflammation beyond the initial cytokine response. Importantly, NF-κB often provides a priming signal for NLRP3 by increasing transcription of NLRP3 and pro-IL-1β. A second activation signal can then be provided by ROS, mitochondrial injury, ATP, or ionic disturbances. This creates a functional TLR4/NF-κB/NLRP3 axis linking extracellular danger recognition with intracellular inflammasome activation. NLRP3 signaling may therefore contribute to myocardial, pulmonary, renal, and endothelial injury following CPB [96,97]. NLRP3 activation requires two signals:

1. Priming Signal (Signal 1): TLR4/NF-κB activation upregulates NLRP3 and pro-IL-1β gene expression [98].
2. Activation Signal (Signal 2): Caused by I/R-mediated ROS, mitochondrial dysfunction, intracellular potassium efflux, or lysosomal rupture [97].

Assembly of the NLRP3 complex cleaves pro-caspase-1 into active caspase-1, which subsequently processes pro-IL-1β and pro-IL-18 into their mature, highly bioactive secreted forms [99]. Active caspase-1 also cleaves Gasdermin D, triggering pyroptosis—an inflammatory programmed cell death pathway that releases intracellular DAMPs into the extracellular milieu [100].

### 5.4. JAK/STAT and Nrf2/ARE Antioxidant Signaling Pathway

The Janus kinase (JAK)/signal transducer and activator of transcription (STAT) pathway provides an important mechanism through which circulating cytokines translate extracellular inflammatory signals into sustained cellular responses [101]. Cytokines such as IL-6, interferons, and several other inflammatory mediators activate membrane-associated cytokine receptors coupled to JAK proteins. Receptor engagement induces JAK phosphorylation and subsequent phosphorylation of STAT proteins. Phosphorylated STATs dimerize and translocate into the nucleus, where they regulate transcription of genes involved in inflammation, immunity, cell survival, and acute-phase responses [101]. During CPB, increased concentrations of IL-6 and other cytokines can therefore activate JAK/STAT signaling in circulating leukocytes and endothelial cells. STAT3 is particularly relevant to IL-6-mediated inflammatory signaling and may contribute to the hepatic acute-phase response and systemic inflammatory phenotype. STAT1 is more closely associated with interferon signaling and inflammatory gene expression. JAK/STAT signaling interacts with NF-κB and MAPK pathways, creating extensive intracellular cross-talk [102]. Persistent activation may contribute to endothelial activation, leukocyte recruitment, and postoperative organ inflammation. Conversely, specific STAT-dependent responses may also participate in tissue protection and resolution, emphasizing the context-dependent nature of this pathway. Thus, JAK/STAT signaling serves as an important intermediary between CPB-induced cytokine release and transcriptional reprogramming in immune and vascular cells [103,104].

The nuclear factor erythroid 2-related factor 2 (Nrf2) pathway is a major endogenous mechanism that counteracts oxidative stress generated during CPB. Nrf2 is normally maintained at low cytoplasmic concentrations through interaction with Kelch-like ECH-associated protein 1 (Keap1), which promotes its ubiquitination and degradation. Increased ROS and electrophilic stress modify critical cysteine residues in Keap1, facilitating Nrf2 stabilization and nuclear translocation [104]. Nuclear Nrf2 binds antioxidant response elements (AREs) within the promoters of numerous cytoprotective genes. These include heme oxygenase-1, NAD(P)H quinone dehydrogenase 1, glutathione-related enzymes, and other antioxidant proteins. CPB creates several sources of oxidative stress, including ischemia–reperfusion, leukocyte activation, mitochondrial dysfunction, and NADPH oxidase activity. Nrf2 activation therefore represents an endogenous compensatory response that limits oxidative damage. However, excessive oxidative stress may overwhelm this protective system or alter the balance between Nrf2-mediated protection and pro-inflammatory pathways. Nrf2 also interacts with NF-κB, potentially suppressing excessive inflammatory transcription while supporting cellular redox homeostasis. The balance between Nrf2-dependent antioxidant responses and ROS-generating pathways may therefore influence the severity of CPB-associated tissue injury. The Nrf2 pathway should consequently be considered an important component of the molecular counter-regulatory response to CPB-induced inflammation [92].

### 5.5. NADPH Oxidase/ROS and Endothelial eNOS/NO Signaling Pathway

NADPH oxidases represent an important source of reactive oxygen species during CPB. Several NOX isoforms are expressed in vascular and immune cells, with NOX2 being particularly relevant to phagocytic respiratory burst, while NOX1 and NOX4 contribute to vascular and cellular redox signaling. CPB-associated inflammatory stimuli, including TLR activation, TNF-α, angiotensin II, mechanical stress, and ischemia–reperfusion, can increase NADPH oxidase activity [105,106]. Increased NOX activity generates superoxide and other ROS that can alter proteins, lipids, DNA, and mitochondrial function. ROS also function as signaling molecules capable of activating NF-κB, MAPK, inflammasomes, and other inflammatory pathways. Evidence indicates that TLR signaling can interact with NOX-dependent ROS production, creating a reciprocal relationship between innate immune activation and oxidative stress. ROS may additionally promote mitochondrial permeability changes and release of mitochondrial DAMPs, further stimulating innate immune pathways. Oxidative stress can impair endothelial nitric oxide bioavailability by promoting NO scavenging and eNOS dysfunction [107]. In leukocytes, ROS contribute to degranulation, NET formation, and inflammatory amplification. Excessive ROS production therefore transforms an initially adaptive redox response into a mechanism of tissue injury. The NOX/ROS pathway represents a critical molecular link connecting CPB, inflammation, endothelial dysfunction, mitochondrial injury, and postoperative organ dysfunction [108,109].

The endothelial nitric oxide synthase (eNOS)/nitric oxide (NO) pathway is a central regulator of vascular tone and endothelial homeostasis and is significantly affected during CPB. Under physiological conditions, eNOS generates NO from L-arginine in endothelial cells, promoting vasodilation, inhibiting platelet activation, and limiting leukocyte adhesion. CPB-associated inflammation can impair eNOS activity through oxidative stress, cytokine signaling, altered shear stress, and reduced tetrahydrobiopterin availability [110]. Oxidative stress may induce eNOS uncoupling, whereby the enzyme produces superoxide rather than NO. Reduced NO bioavailability facilitates vasoconstriction, platelet activation, leukocyte adhesion, and endothelial dysfunction. At the same time, inflammatory cytokines and NF-κB activation can upregulate inducible nitric oxide synthase (iNOS), producing large amounts of NO [111,112]. Excessive NO, in the presence of superoxide, can generate peroxynitrite, a highly reactive oxidant that damages proteins and membranes. Thus, CPB-associated inflammation can shift the vascular redox balance from eNOS-derived protective NO toward oxidative and inflammatory signaling. This mechanism is particularly relevant to vasoplegia, microcirculatory dysfunction, and endothelial injury after CPB. The interaction between NO, ROS, NF-κB, and mitochondrial function represents an important example of cross-talk between vascular and inflammatory signaling pathways [113,114].

### 5.6. Leukocyte Adhesion: Selectin/Integrin–PLCγ2/Akt/p38 MAPK Signaling and Overall Molecular Cascade During CPB

Leukocyte recruitment during CPB follows a coordinated sequence of rolling, activation, adhesion, and transmigration. Endothelial activation increases the expression or surface availability of adhesion molecules such as P-selectin, E-selectin, ICAM-1, and VCAM-1. Selectin engagement initiates intracellular signaling in leukocytes involving PLCγ2, p38 MAPK, and Akt [115,116]. Chemokines presented on the endothelial surface then activate leukocyte G-protein-coupled receptors and induce conformational activation of β2 integrins. Activated integrins bind endothelial ICAM-1 and VCAM-1, producing firm adhesion. Subsequent cytoskeletal rearrangement allows leukocytes to migrate across the endothelial barrier. CPB modifies this process by altering intracellular phosphorylation and leukocyte responsiveness. Experimental evidence indicates that CPB can reduce selectin-mediated slow rolling while increasing chemokine-induced neutrophil arrest and transmigration. These changes may contribute to excessive neutrophil recruitment into tissues after CPB. Activated neutrophils release proteases, ROS, myeloperoxidase, and other mediators capable of damaging the endothelial barrier. Thus, adhesion signaling is a crucial cellular amplification step linking circulating inflammatory mediators to tissue infiltration and postoperative organ injury [117].

The molecular response to CPB can therefore be conceptualized as a multidirectional network rather than a single signaling pathway. Contact between blood and the extracorporeal circuit activates complement, coagulation, platelets, and leukocytes, while surgical trauma and ischemia–reperfusion generate endogenous danger signals. These signals activate PRRs, particularly TLR4 and other innate immune receptors, which converge on NF-κB and MAPK signaling. At the same time, PI3K/Akt/mTOR, JAK/STAT, and endothelial signaling pathways modulate cellular survival, metabolism, cytokine responses, and vascular function. Oxidative stress generated by NOX enzymes and dysfunctional mitochondria further amplifies inflammatory signaling and promotes NLRP3 inflammasome activation. Inflammatory cytokines subsequently activate additional endothelial and leukocyte signaling pathways, resulting in adhesion, transmigration, and tissue infiltration [118]. The resulting interaction between immune activation, oxidative stress, endothelial dysfunction, and mitochondrial injury can propagate systemic inflammation beyond the period of CPB itself. Counter-regulatory mechanisms, including IL-10, Nrf2, PI3K/Akt-dependent survival pathways, and other resolution mechanisms, attempt to restore homeostasis. When these compensatory mechanisms are insufficient, the inflammatory response may become maladaptive and contribute to vasoplegia, myocardial injury, acute kidney injury, pulmonary dysfunction, neurological complications, and multiple-organ dysfunction. Understanding these interconnected pathways is therefore essential for developing targeted strategies to attenuate the maladaptive inflammatory response while preserving the physiological immune and repair functions required after cardiac surgery [2].

## 6. End-Organ Pathophysiology and Clinical Manifestations

The convergence of humoral activation, leukocyte infiltration, intracellular signaling, and microvascular barrier disruption leads to organ dysfunction [119].

### 6.1. Vasoplegic Shock and Cardiovascular Collapse

Vasoplegic syndrome affects 5% to 25% of patients following CPB, characterized by severe hypotension, low systemic vascular resistance (SVR), and normal or elevated cardiac index [120,121]. Low cardiac output syndrome (LCOS) and severe, profound left ventricular hypoperfusion-related dysfunction are independent risk factors that significantly influence the outcomes of surgical procedures. Cardiopulmonary bypass (CPB) and the induction of cardioplegic cardiac arrest during coronary artery surgery in these patients may represent additional independent risk factors. Experimental studies have demonstrated that cardiodepressive mediators are released during reperfusion, resulting in the production of pro-inflammatory cytokines [122]. Cardiac arrest during surgery on the arrested heart, in which native coronary circulation is interrupted, inevitably results in a period of myocardial ischemia. During ischemia, vasodilatory metabolites, including potassium, hydrogen ions, lactate, pyruvate, and adenosine, are generated as a consequence of anaerobic metabolism. These mediators induce arteriolar vasodilation throughout the ischemic period. Once coronary bypass grafting is complete and myocardial blood flow is restored, hyperperfusion occurs through the previously dilated vascular bed, increasing intravascular volume within the myocardium. At the same time, autoregulatory mechanisms become impaired. Consequently, blood flow, including fluid movement across the capillary–interstitial interface, becomes largely dependent on pressure gradients. This may result in capillary wall injury, increased vascular permeability, and transudation of fluid into the interstitial space, clinically manifested as myocardial edema [120,122].

During the ischemic period, myocardial cells become injured and increasingly depend on anaerobic metabolism. After reperfusion and exposure to hyperoxia, cells are suddenly exposed to excess oxygen that the compromised mitochondrial machinery cannot efficiently utilize. Lipid metabolism is subsequently disturbed, particularly within cellular membranes. This process promotes the formation of reactive oxygen species (ROS) and free radicals. These molecules contain one or more unpaired electrons in their outer orbital, rendering them highly reactive. Important reactive species include the superoxide radical, peroxide anion, hydrogen peroxide, hydroxyl radical, lipid radicals, and peroxyl radicals. Their unpaired electrons confer high chemical reactivity, leading to disruption of cellular homeostasis and direct cellular injury through lipid peroxidation. Consequently, oxidative injury may contribute substantially to myocardial damage during reperfusion [123].

Local myocardial cooling and myocardial protection with cardioplegic solutions are therefore essential to minimize ischemic injury during cardiac arrest and to attenuate the subsequent ischemia–reperfusion syndrome. However, in patients with severe left ventricular dysfunction, the geometry of the affected ventricle may be altered, while the coronary arteries may demonstrate extensive and severe obstructive or stenotic lesions [124]. These conditions may result in heterogeneous and suboptimal distribution of the cardioplegic solution, potentially compromising myocardial protection in an already severely vulnerable myocardium.

**Figure 4:**
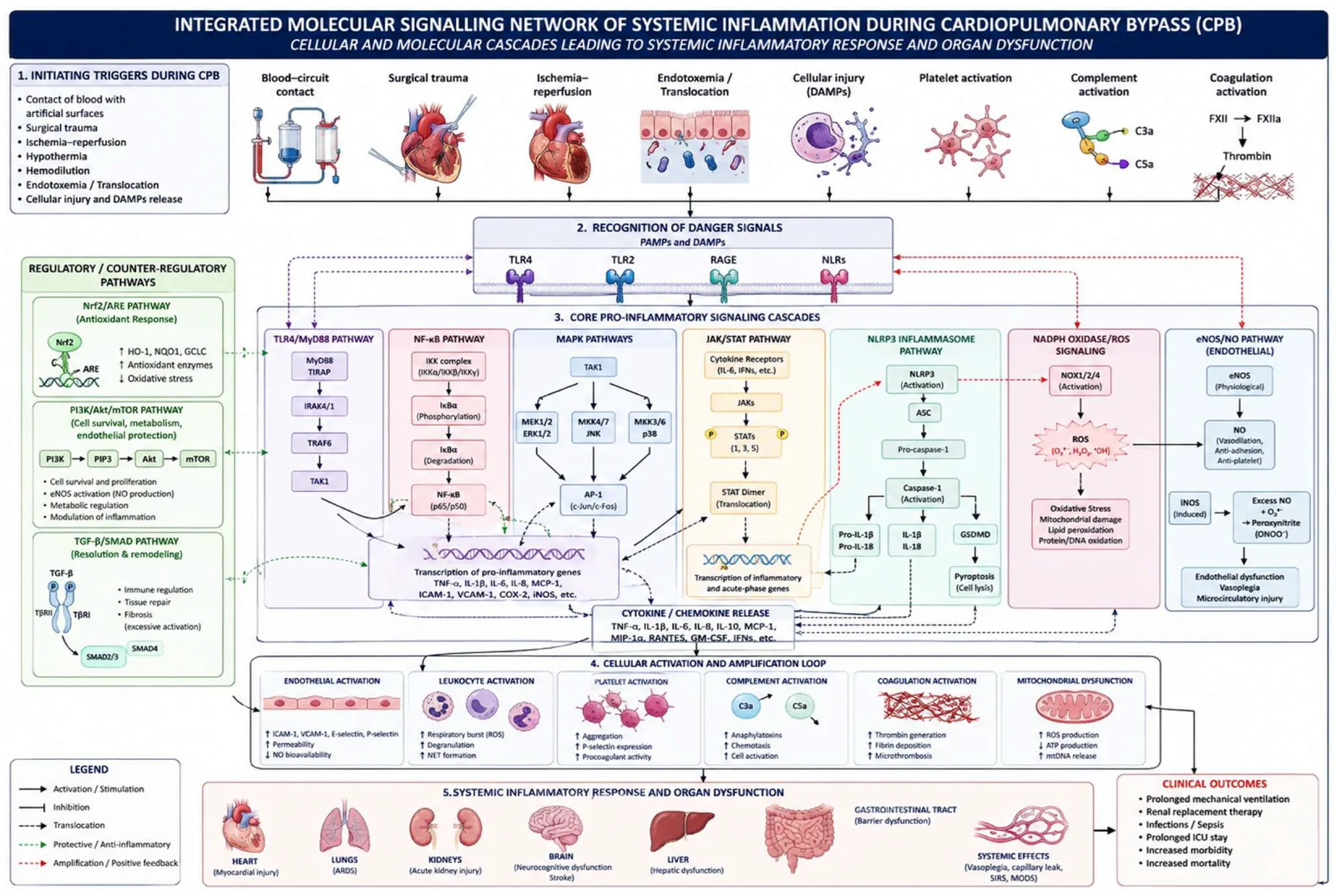
Integrated molecular signaling network of systemic inflammation during cardiopulmonary bypass (CPB). CPB-related triggers—including blood–surface interaction, surgical trauma, ischemia–reperfusion injury, endotoxemia, cellular damage, platelet activation, complement activation, and coagulation activation—initiate PAMP/DAMP recognition and activate interconnected TLR4/MyD88, NF-κB, MAPK, JAK/STAT, NLRP3 inflammasome, NADPH oxidase/ROS, and endothelial eNOS/NO pathways. These signaling cascades promote cytokine release, endothelial and leukocyte activation, oxidative and mitochondrial injury, and amplification of the systemic inflammatory response, ultimately contributing to postoperative organ dysfunction and adverse clinical outcomes.

Pathophysiological drivers include the following:

- Overproduction of nitric oxide (NO) via upregulated inducible NO synthase (iNOS) [122].
- Activation of vascular smooth muscle ATP-sensitive potassium channels (K-{ATP}), leading to hyperpolarization and vascular unresponsiveness to endogenous catecholamines [123].
- Depletion of endogenous vasopressin stores from the posterior pituitary [124].
- Myocardial stunning mediated by TNF-α, IL-6, and reperfusion-induced mitochondrial calcium overload (Figure 4) [125].

### 6.2. Acute Lung Injury (ALI) and ARDS

The lungs act as a primary target organ for CPB-induced inflammatory injury [126]. Re-ventilation following pulmonary ischemia causes massive pulmonary sequestration of activated neutrophils [127]. Neutrophil degranulation, ROS production, and NETosis damage alveolar epithelial and pulmonary capillary endothelial cells [128]. Degradation of the alveolar-capillary barrier leads to protein-rich fluid extravasation, loss of surfactant activity, reduced lung compliance, atelectasis, and severe ventilation-perfusion mismatch, culminating in post-perfusion lung syndrome or ARDS [129].

Patients undergoing cardiopulmonary bypass may experience a variable degree of postoperative pulmonary dysfunction. Although pulmonary impairment remains subclinical in the majority of patients, approximately 10% of patients undergoing cardiac surgery develop clinically significant respiratory insufficiency. A comprehensive review of patients undergoing coronary surgery demonstrated that acute respiratory distress syndrome (ARDS) developed in approximately 2% of cases during the immediate postoperative period [126].

The classical manifestations of postoperative pulmonary dysfunction include reduced arterial oxygen saturation, decreased static and dynamic pulmonary compliance, and increased pulmonary vascular resistance. Alterations in pulmonary capillary permeability contribute substantially to hypoxemia and reduced pulmonary compliance, as water and plasma proteins extravasate into the pulmonary interstitium and alveolar spaces. The resulting inactivation and dysfunction of pulmonary surfactant promote localized areas of atelectasis and increase intrapulmonary right-to-left shunting. Similar mechanisms underlying respiratory dysfunction after cardiac surgery and CPB contribute to the pathophysiology of ARDS. Pro-inflammatory cascades, including activation of the complement system and release of cytokines—particularly tumor necrosis factor-alpha (TNF-α), interleukin-6 (IL-6), interleukin-8 (IL-8), and elastase—promote disruption of pulmonary capillary permeability and endothelial integrity. These mediators contribute to pulmonary edema, impaired gas exchange, neutrophil accumulation, and progressive pulmonary tissue injury [128].

Even in patients who are not directly exposed to CPB, several mechanisms may contribute to postoperative pulmonary complications secondary to the surgical procedure itself. These include direct surgical trauma, median sternotomy, opening of the pleural spaces, and local myocardial cooling with ice. Nevertheless, CPB represents an important additional risk factor for postoperative pulmonary complications. Such complications may be associated with blood exposure to artificial surfaces, hypothermia, inflammatory activation, and, particularly, ischemia–reperfusion injury involving the heart and lungs [127,128].

### 6.3. Cardiac Surgery-Associated Acute Kidney Injury (CSA-AKI)

CSA-AKI occurs in up to 30% of high-risk patients and independently increases perioperative mortality [130]. Pathophysiological mechanisms include the following:

- Renal medullary hypoxia secondary to non-pulsatile flow and impaired autoregulation [131].
- Hemolysis-induced release of cell-free hemoglobin, which scavenges NO, induces renal vasoconstriction, and exerts direct cytotoxicity on proximal tubular epithelial cells via Fenton reactions [132].
- Microvascular congestion driven by endothelial swelling, NET formation, and platelet–leukocyte microthrombi [133].

Cardiopulmonary bypass induces a transient impairment of renal function. In patients with normal preoperative renal function, most patients develop only subclinical renal dysfunction, whereas less than 1% develop clinically apparent renal failure [130]. Several potential etiological mechanisms may contribute to the development of acute kidney injury (AKI) during cardiac surgery requiring CPB, including non-pulsatile flow, release of inflammatory mediators such as endothelin and elastase, generation of free radicals, hypothermia, renal hypoperfusion, and hemolysis with the release of free hemoglobin from mechanically damaged erythrocytes.

The duration of CPB is directly correlated with the incidence and severity of postoperative renal dysfunction in patients undergoing cardiac surgery [132]. Renal dysfunction represents one of the major causes of morbidity and mortality following CPB, particularly in patients with pre-existing renal impairment and in those who develop significant postoperative kidney injury.

Patients with pre-existing mild renal dysfunction have approximately a fourfold increased risk of developing postoperative renal failure requiring dialysis compared with patients with normal baseline renal function. The reported mortality among patients who develop postoperative renal failure requiring dialysis ranges from approximately 7% to 38% [130].

From a mechanistic perspective, CPB-associated renal injury is multifactorial and involves the interaction between reduced renal perfusion, systemic inflammatory activation, oxidative stress, hemolysis, microembolization, neurohormonal activation, and endothelial dysfunction. Inflammatory mediators and free hemoglobin may further promote tubular epithelial injury, mitochondrial dysfunction, and intrarenal microvascular impairment, thereby contributing to the progression from subclinical renal dysfunction to clinically significant AKI [132].

### 6.4. Neuroinflammatory Sequelae

Postoperative cognitive dysfunction (POCD) and encephalopathy stem from a combination of microembolism (gaseous and particulate) and systemic inflammation [134]. Systemic pro-inflammatory cytokines breach the blood–brain barrier (BBB) by disrupting endothelial tight junction proteins (ZO-1, occludin) [135]. Microglial activation within the central nervous system leads to local neuroinflammation, synaptic dysfunction, and neurocognitive impairment [136]. The risk of stroke following conventional cardiac surgery is approximately 1% among patients undergoing surgery during their sixth decade of life and approximately doubles with each subsequent decade of age. Major risk factors for the development of postoperative neurological complications include a previous cerebrovascular event, impaired left ventricular function, unstable angina, and emergency surgery [135].

The occurrence of a postoperative neurological complication is associated with prolonged hospitalization, increased intensive care unit (ICU) length of stay, and substantial mortality. In patients who develop major neurological complications, the duration of ICU hospitalization may increase from an average of 2–3 days to approximately 25 days, with mortality rates approaching 20%. Neurological complications account for approximately 18–25% of fatal postoperative events following conventional cardiac surgery [136]. The key mechanisms underlying neurological events associated with cardiac surgery and CPB include cerebral microembolization, particularly from manipulation of the ascending aorta. Additional potential mechanisms of cerebral injury following conventional CABG include periods of reduced mean arterial pressure during non-pulsatile CPB, impaired cerebral autoregulation, increased extracellular fluid within the intracranial compartment, and the formation or passage of microscopic air bubbles. In addition, particulate emboli originating from atherosclerotic aortic plaques, thrombotic material, platelet aggregates, and gaseous microemboli may contribute to cerebral microvascular obstruction and neuronal injury.

Collectively, these mechanisms demonstrate that postoperative neurological injury after CPB is multifactorial, involving embolic phenomena, hypoperfusion, inflammatory activation, endothelial dysfunction, and ischemia–reperfusion-related oxidative stress. The interaction between these mechanisms may be particularly important in elderly patients and in individuals with advanced aortic atherosclerosis or pre-existing cerebrovascular disease [134].

## 7. Therapeutic Strategies and Future Interventions

Addressing CPB-induced SIRS requires a targeted approach combining technological modifications and pharmacological therapies [137].

### 7.1. Circuit Engineering and Biocompatibility

Advances in surface chemistry have yielded biocompatible circuit coatings categorized into heparin-based (e.g., Carmeda BioActive Surface), synthetic non-thrombogenic polymers (e.g., poly-methoxyethyl acrylate [PMEA]), and biopassive surfaces [138,139]. These coatings reduce plasma protein adsorption, blunt Factor XII autoactivation, and attenuate complement cleavage [140].

Miniaturized Extracorporeal Circuits (MeCC) further suppress inflammation by removing the venous reservoir, eliminating the air–blood interface, and significantly reducing circuit priming volume, thereby minimizing hemodilution and contact activation (Table 3) [141,142].

### 7.2. Hemoadsorption Techniques

Extracorporeal cytokine adsorption using broad-spectrum polymer adsorbents (e.g., CytoSorb^®^) integrated into the CPB circuit offers a therapeutic modality to clear pro-inflammatory mediators [143,144]. By capturing cytokines, free hemoglobin, myoglobin, and activated DAMPs via hydrophobic interactions, hemoadsorption helps stabilize hemodynamic parameters and reduces post-perfusion vasoplegia in high-risk patients [145,146].

### 7.3. Targeted Pharmacological Interventions

1. Corticosteroids: Prophylactic administration of high-dose corticosteroids (e.g., methylprednisolone, dexamethasone) inhibits NF-κB nuclear translocation and reduces perioperative cytokine levels [147]. However, large randomized controlled trials (DECS, SIRS) demonstrated that while corticosteroids suppress inflammatory biomarkers, they do not significantly reduce 30-day mortality and may increase myocardial injury risks in select subgroups [148,149].
2. Complement Interception: Monoclonal antibodies targeting C5 (e.g., eculizumab) and recombinant C1 esterase inhibitors (C1-INH) prevent generation of C5a and C5b-9, effectively reducing pulmonary and myocardial damage in pre-clinical and early clinical trials [150,151].
3. NETosis Regulation: Novel therapeutic strategies aiming to dismantle extracellular DNA traps using intravenous recombinant human DNase I (rhDNase) or inhibiting peptidylarginine deiminase 4 (PAD4)—the enzyme required for histone citrullination—show promise in mitigating microvascular thrombosis and organ dysfunction [152,153].

## 8. Clinical Phenotypes at Risk, Postoperative Outcomes, and Emerging Therapeutic Strategies

### 8.1. Patient Phenotypes at Risk for Exaggerated SIRS, ARDS, and Vasoplegia

The systemic inflammatory response induced by cardiopulmonary bypass (CPB) is highly heterogeneous, and its clinical expression varies substantially among patients. Although a transient inflammatory response is expected after cardiac surgery, a subset of patients develops an exaggerated or persistent inflammatory phenotype characterized by prolonged systemic inflammatory response syndrome (SIRS), endothelial dysfunction, vasoplegia, acute respiratory distress syndrome (ARDS), and multiorgan dysfunction. This heterogeneity suggests that postoperative inflammatory complications result from the interaction between pre-existing patient vulnerability, the magnitude of the surgical insult, CPB-related exposure, transfusion, ischemia–reperfusion injury, and the early postoperative inflammatory and endothelial response.

Several clinical characteristics appear to identify patients with increased susceptibility to an exaggerated inflammatory response. Advanced age, reduced ventricular function, chronic kidney disease, diabetes mellitus, chronic pulmonary disease, obesity, anemia, pre-existing systemic inflammation, and hemodynamic instability may reduce physiological reserve and increase vulnerability to CPB-associated inflammatory injury. In addition, complex or combined procedures, prolonged CPB duration, increased blood-product exposure, and emergency surgery may further amplify the inflammatory response. Recent observational data support the clinical relevance of this phenotype. In a cohort of 1908 patients undergoing cardiac surgery with CPB, postoperative SIRS was observed in 28.7% of patients and was independently associated with increased 30-day mortality [154].

A particularly vulnerable phenotype appears to be represented by patients who develop persistent rather than transient SIRS. In a prospective observational study of 261 patients undergoing cardiac surgery with CPB, prolonged SIRS, defined as fulfillment of at least two SIRS criteria on four consecutive postoperative days, occurred in 6.4% of patients. Prolonged SIRS was independently associated with postoperative atrial fibrillation, 90-day stroke, and 90-day mortality, suggesting that persistence of the inflammatory response may be more clinically relevant than an isolated early postoperative increase in inflammatory markers [155].

ARDS represents another severe manifestation of excessive pulmonary and systemic inflammation after cardiac surgery. The development of postoperative ARDS appears to reflect a multifactorial interaction between pre-existing pulmonary and cardiovascular vulnerability and perioperative inflammatory, mechanical, and transfusion-related insults. Patients with previous cardiac surgery, complex procedures, emergency surgery, pre-existing pulmonary disease, low cardiac output, shock, and substantial exposure to blood products have been reported to have a higher risk of postoperative ARDS. In a cohort of 3946 cardiac surgical patients, previous cardiac surgery, complex surgery, emergency procedures, and transfusion of more than three units of packed red blood cells were identified as independent predictors of ARDS. Earlier studies similarly identified previous cardiac surgery, postoperative shock, and blood-product exposure as important predictors [156].

The association between CPB and pulmonary injury is biologically plausible because extracorporeal circulation activates complement, leukocytes, platelets, and endothelial cells and promotes cytokine release, oxidative stress, leukocyte adhesion, and microvascular injury. These mechanisms may increase pulmonary endothelial permeability and contribute to interstitial and alveolar edema. Older age, heart failure, emergency surgery, transfusion requirements, and prolonged CPB duration may further increase the risk of pulmonary complications.

Vasoplegia represents a distinct but closely related inflammatory phenotype characterized by profound loss of vascular tone, reduced systemic vascular resistance, and hypotension that may persist despite adequate or increased cardiac output. The pathophysiology involves excessive nitric oxide (NO) production, activation of soluble guanylate cyclase, ATP-sensitive potassium channels, inflammatory signaling, endothelial dysfunction, and relative vasopressin deficiency. Current evidence indicates that susceptibility to postoperative vasoplegia is influenced by both patient-related and procedure-related factors. Advanced age, reduced left ventricular ejection fraction, heart failure, chronic kidney disease, anemia, diabetes mellitus, infective endocarditis, preoperative hemodynamic instability, and treatment with renin–angiotensin–aldosterone system inhibitors have been associated with increased risk. Intraoperative contributors include CPB exposure, blood-product transfusion, temperature disturbances, and complex cardiac procedures [157].

The treatment of refractory vasoplegia after CPB has increasingly focused on pharmacological strategies targeting the excessive NO-mediated vasodilatory state. Among these approaches, hydroxocobalamin, a form of vitamin B12, has emerged as a potential rescue therapy for patients with severe vasoplegia refractory to conventional vasopressors.

The rationale for hydroxocobalamin is closely related to the molecular mechanisms underlying vasoplegia. Excessive inducible nitric oxide synthase activity and NO production contribute to activation of soluble guanylate cyclase, increased cyclic guanosine monophosphate signaling, vascular smooth muscle relaxation, and profound reduction in systemic vascular resistance. Hydroxocobalamin is capable of binding NO and other gaseous mediators and may therefore attenuate NO-dependent vasodilation and restore vascular tone [158].

Initial clinical evidence consisted predominantly of case reports and retrospective case series. In a case series of 33 cardiac surgical patients with refractory vasoplegia during or immediately after CPB, intravenous hydroxocobalamin was associated with an increase in mean arterial pressure and a reduction in vasopressor requirements in a substantial proportion of patients. However, the absence of randomized controls and the potential for publication and selection bias substantially limit conclusions regarding efficacy.

More recent evidence provides somewhat stronger support for a hemodynamic effect. A systematic review and meta-analysis comparing hydroxocobalamin with methylene blue in cardiac surgical patients with vasoplegic shock included 263 patients from four retrospective observational studies. Hydroxocobalamin was associated with a greater increase in mean arterial pressure at one hour and lower vasopressor requirements at one and six hours, although no significant difference in mortality was observed. Importantly, the authors emphasized that the available evidence was limited by the observational design and risk of bias and that adequately powered randomized trials are still required [159].

Therefore, hydroxocobalamin should currently be considered an adjunctive or rescue therapy for severe vasoplegia refractory to conventional vasopressor therapy, rather than a replacement for norepinephrine or vasopressin. A commonly reported regimen is 5 g administered intravenously over approximately 15 min, although this dosing strategy has largely been extrapolated from its established use in cyanide toxicity, and no dedicated dose-finding trial has definitively established the optimal dose or duration of treatment for vasoplegia [160].

The use of hydroxocobalamin is also associated with clinically relevant practical considerations. Its intense red coloration can cause transient discoloration of plasma and urine and may interfere with selected laboratory measurements. It may also interfere with certain extracorporeal blood purification systems, which is particularly important in cardiac surgical patients who develop concurrent AKI and require renal replacement therapy. These considerations should be incorporated into clinical decision-making.

It is important to distinguish hydroxocobalamin from methylcobalamin. Although both are forms of vitamin B12, the clinical literature supporting treatment of post-CPB vasoplegia predominantly concerns hydroxocobalamin. Current evidence supporting methylcobalamin as a therapeutic agent for vasoplegia is extremely limited, and there is insufficient clinical evidence to recommend methylcobalamin as a standard treatment. Accordingly, hydroxocobalamin, rather than methylcobalamin, should be considered the clinically relevant vitamin B12 derivative in the context of refractory post-CPB vasoplegia.

Overall, hydroxocobalamin represents a biologically plausible and increasingly used rescue therapy for refractory vasoplegia, but its current evidence base remains substantially weaker than that supporting conventional vasopressor therapy. Future randomized studies should determine the optimal timing, dose, patient selection, comparative efficacy, and effects on organ dysfunction and survival.

Importantly, these risk factors should not be interpreted as deterministic predictors. Rather, SIRS, ARDS, and vasoplegia appear to represent overlapping clinical manifestations of a broader high-risk inflammatory and endothelial phenotype, in which preoperative vulnerability interacts with the magnitude and duration of the inflammatory stimulus generated by surgery and CPB.

While a transient inflammatory response is an expected component of recovery from cardiac surgery, persistent or excessive inflammation may become clinically detrimental. Prolonged SIRS has been associated with postoperative organ dysfunction, prolonged intensive care unit (ICU) and hospital stay, respiratory complications, acute kidney injury (AKI), atrial arrhythmias, neurological complications, and increased mortality.

In the prospective study by Viikinkoski et al., prolonged SIRS occurred in 6.4% of patients and was independently associated with postoperative atrial fibrillation, 90-day stroke, and mortality [154]. The reported odds ratio for 90-day mortality was 10.7, although the relatively small number of patients with prolonged SIRS resulted in wide confidence intervals. These findings suggest that persistent inflammation is not merely an epiphenomenon of complicated postoperative recovery but may represent an important marker of systemic physiological deterioration.

Similarly, a larger cohort of 1908 patients demonstrated a strong association between early postoperative SIRS and 30-day mortality. Mortality was 12.2% among patients with SIRS compared with 1.5% among those without SIRS. After adjustment and propensity-score matching, SIRS remained independently associated with 30-day mortality, with an odds ratio of 2.77 (95% CI, 1.40–5.47). These findings reinforce the concept that the magnitude of postoperative inflammation may provide prognostic information beyond traditional surgical risk variables [154].

The clinical consequences of vasoplegia are similarly determined by its severity and duration. Persistent vasodilatory shock may lead to inadequate tissue perfusion despite apparently preserved cardiac output and may contribute to renal, neurological, hepatic, and gastrointestinal dysfunction. Vasoplegia is particularly concerning when it coexists with low cardiac output, acute kidney injury, respiratory failure, or other manifestations of multiorgan dysfunction. Contemporary reviews consistently associate clinically significant vasoplegia after cardiac surgery with increased morbidity and mortality, although considerable heterogeneity exists in diagnostic criteria, treatment strategies, and patient populations.

Importantly, the association between SIRS or vasoplegia and mortality should not necessarily be interpreted as a direct causal relationship. Severe inflammation and vasoplegia frequently occur in patients undergoing complex surgery and may coexist with bleeding, low cardiac output syndrome, infection, AKI, respiratory failure, and other complications. Thus, prolonged SIRS and vasoplegia may function both as mediators of organ injury and markers of overall disease severity. Nevertheless, recognition of these phenotypes at an early stage may facilitate closer hemodynamic monitoring, organ-protective strategies, and timely escalation of supportive treatment [161].

### 8.2. SGLT2 Inhibition and Renal Protection After Cardiac Surgery

Sodium-glucose cotransporter-2 (SGLT2) inhibitors have recently emerged as a potential pharmacological strategy for prevention of cardiac surgery-associated acute kidney injury (CSA-AKI). Their potential renoprotective effects may involve restoration of tubuloglomerular feedback, reduction in intraglomerular pressure, modulation of tubular oxygen consumption and metabolic substrate utilization, and attenuation of oxidative and inflammatory pathways. These mechanisms are particularly relevant in the setting of CPB, in which renal injury may result from the combined effects of systemic inflammation, hemodilution, altered renal perfusion, hemolysis, oxidative stress, and microvascular dysfunction.

Early perioperative clinical evidence was limited but encouraging. In an open-label randomized pilot study of 55 patients undergoing elective cardiac surgery with CPB, perioperative empagliflozin administration was not associated with significant reductions in NGAL or KIM-1 concentrations, but the incidence of AKI during the first seven postoperative days was substantially lower with empagliflozin than with standard care (20% versus 66.7%). The investigators appropriately emphasized that these findings were hypothesis-generating and required confirmation in larger randomized trials [162].

More recently, this evidence was strengthened by the multicenter MERCURI-2 randomized clinical trial. In this study, 784 patients undergoing elective cardiac surgery were randomized to receive dapagliflozin 10 mg or placebo beginning one day before surgery and continuing through the second postoperative day. AKI during the first seven postoperative days occurred in 28% of patients receiving dapagliflozin compared with 52% receiving placebo, corresponding to a relative risk of 0.54 (95% CI, 0.45–0.65; *p* < 0.001) [163].

These findings provide the strongest evidence to date that short-term perioperative SGLT2 inhibition may reduce AKI following elective cardiac surgery. However, several considerations remain important. First, the population consisted of elective cardiac surgical patients, and the extent to which the findings can be generalized to emergency surgery, severe hemodynamic instability, advanced chronic kidney disease, or other high-risk CPB populations remains uncertain. Second, although the trial provides strong evidence for reduction in AKI, it does not establish that SGLT2 inhibition improves long-term renal function, dialysis-free survival, or mortality. Finally, perioperative SGLT2 inhibitor therapy must be balanced against the potential risks of euglycemic ketoacidosis, ketosis, volume depletion, and metabolic instability during fasting and surgical stress.

Thus, SGLT2 inhibition represents a promising emerging component of renal-protective strategies in cardiac surgery, but its optimal perioperative timing, patient selection, and integration with established hemodynamic and organ-protective measures require further investigation. Several pharmacological approaches have previously been investigated for the prevention of CSA-AKI, including nesiritide and fenoldopam. Although both agents have biologically plausible renal-protective mechanisms, clinical evidence has not established either drug as a standard strategy for renal protection during CPB [164].

Nesiritide, a recombinant form of B-type natriuretic peptide, promotes natriuresis and vasodilation and may improve renal perfusion while suppressing components of the renin–angiotensin–aldosterone system. In the NAPA trial, which included patients with left ventricular dysfunction undergoing CABG with CPB, perioperative nesiritide was associated with a smaller postoperative increase in serum creatinine, a smaller decline in glomerular filtration rate, and increased urine output. A possible reduction in 180-day mortality was also reported [165].

However, subsequent prospective randomized evidence did not confirm a clinically meaningful benefit on hard renal outcomes. In a randomized trial of high-risk cardiac surgery patients, prophylactic nesiritide did not reduce the combined incidence of dialysis or death despite a reduction in early postoperative AKI and serum creatinine. Therefore, the available evidence suggests that nesiritide may improve selected early renal physiological parameters without demonstrating a consistent reduction in dialysis, mortality, or other major clinical outcomes.

Fenoldopam, a selective dopamine-1 receptor agonist, produces renal and systemic vasodilation and was initially considered attractive for improving renal blood flow during cardiac surgery. Early randomized studies suggested potential reductions in AKI among selected high-risk patients. However, these findings were not consistently reproduced in larger studies.

Most importantly, the multicenter randomized trial by Bove et al. included 667 patients who developed AKI after cardiac surgery. Fenoldopam did not reduce the need for renal replacement therapy compared with placebo (20% versus 18%; *p* = 0.47) and did not reduce 30-day mortality. Moreover, hypotension occurred more frequently with fenoldopam (26% versus 15%; *p* = 0.001), which is particularly relevant in patients already vulnerable to vasoplegia and impaired organ perfusion [166].

Taken together, current evidence does not support the routine prophylactic use of either nesiritide or fenoldopam as standard renal-protective therapy during CPB. Their historical importance remains relevant because these studies illustrate the difficulty of translating improvements in renal blood flow or surrogate biomarkers into reductions in clinically meaningful outcomes. Current renal-protection strategies should instead emphasize optimization of systemic perfusion and oxygen delivery, avoidance of nephrotoxins, individualized fluid management, prevention of excessive hemodilution and transfusion, and early recognition and management of AKI. Pharmacological interventions, such as SGLT2 inhibition, may represent a new direction, but further evidence is required before they can be incorporated universally into perioperative protocols.

## 9. Conclusions and Future Directions

The systemic inflammatory response during coronary artery bypass grafting with cardiopulmonary bypass remains a major contributor to postoperative morbidity and organ injury. CPB triggers a complex pathophysiology characterized by the following:

1. Rapid contact activation of humoral cascades (complement, contact, coagulation).
2. Endothelial glycocalyx degradation and cellular hyperactivation (neutrophils, monocytes, platelets).
3. Activation of intracellular molecular axes, including TLR4/MyD88, NF-κB, MAPK, and NLRP3 inflammasome pathways, driving a cytokine storm.
4. Microvascular leakage, NETosis, vasoplegia, and multi-organ damage.

While advances in biocompatible surfaces, miniaturized circuits, and hemoadsorption technologies have improved perioperative safety, pharmacologic strategies require refined targeting. Future progress lies in personalized immunomodulation—utilizing real-time point-of-care biomarker profiling (e.g., IL-6, C5a, citrullinated histone H3) to identify patients at high risk for hyperinflammation and deploying targeted therapies tailored to individual inflammatory phenotypes.

## Figures and Tables

**Table 1:** Major cellular components contributing to the systemic inflammatory response during cardiopulmonary bypass (CPB), including neutrophils, monocytes/macrophages, endothelial cells, platelets, complement, and dendritic cells. Principal CPB-related triggers, activated receptors and pathways, inflammatory mediators, and their effects on vascular and tissue injury are indicated.

Cell Type	Major Triggers During CPB	Activated Receptors/Pathways	Key Mediators Released	Principal Effects
Neutrophils	Blood–air interface, ischemia–reperfusion, shear stress, complement (C5a), fMLP	TLR4, Complement receptors (CR1, CR3), FcγR	ROS, elastase, myeloperoxidase (MPO), NETs, IL-8, TNF-α	Endothelial injury, microvascular dysfunction, tissue damage
Monocytes/Macrophages	Endotoxin, DAMPs, cytokines (IL-1β, TNF-α), hypoperfusion	TLR2, TLR4, NLRP3	IL-1β, IL-6, TNF-α, IL-10, MCP-1, tissue factor	Amplification of inflammation, coagulation activation
Endothelial Cells	Shear stress, hypothermia, ischemia–reperfusion, complement activation	TLR4, PAR-1, NLRP3	IL-6, IL-8, ICAM-1, VCAM-1, E-selectin, vWF, NO	Leukocyte adhesion, increased permeability, procoagulant state
Platelets	Contact activation, shear stress, thrombin	TLR4, PAR receptors, GPVI	PF4, CD40L, serotonin, TXA_2_, P-selectin, microparticles	Leukocyte recruitment, coagulation, endothelial activation
Complement System	Contact activation, endotoxin, immune complexes	Classical, lectin and alternative pathways	C3a, C5a, MAC (C5b-9)	Anaphylatoxin release, leukocyte activation, cell lysis
Dendritic Cells	DAMPs, cytokines, ischemia	TLR2, TLR4, CD40	IL-12, IL-23, type I IFNs, co-stimulatory molecules	Activation of T cells, promotion of adaptive immunity

**Table 2:** Key molecular mediators and signaling pathways involved in CPB-induced systemic inflammation. Major activation stimuli, intracellular signaling molecules, downstream inflammatory effects, and potential physiological and clinical consequences, including endothelial dysfunction, microvascular injury, and organ dysfunction, are outlined. ↑ indicates increased levels or activity.

Pathway/Mediator	Activation Stimuli During CPB	Key Signaling Molecules	Downstream Effects	Physiological/Clinical Consequences
Toll-like receptor (TLR) signaling	Endotoxin, DAMPs, ischemia–reperfusion	TLR2, TLR4, MyD88, TRIF, NF-κB, IRF3	↑ Pro-inflammatory cytokines (TNF-α, IL-1β, IL-6), type I IFNs	SIRS, endothelial activation, organ dysfunction
NF-κB pathway	Cytokines, ROS, mechanical stress	IKK complex, IκBα, NF-κB (p65/p50)	↑ TNF-α, IL-1β, IL-6, IL-8, ICAM-1, VCAM-1	Systemic inflammation, leukocyte recruitment
NLRP3 inflammasome	DAMPs, ROS, ionic flux (K^+^ efflux), lysosomal damage	NLRP3, ASC, caspase-1	↑ IL-1β, IL-18, pyroptosis	Amplification of SIRS, tissue injury
Complement cascade	Contact activation, endotoxin, immune complexes	C3, C5, C3a, C5a, C5b-9 (MAC)	Anaphylatoxins (C3a, C5a), cell lysis, chemotaxis	Vasodilation, increased permeability, organ injury
Coagulation system	Contact activation, tissue factor release, hypoperfusion	Thrombin, Factor Xa, Fibrin, TAFI	Fibrin formation, platelet activation, microthrombosis	Microvascular dysfunction, organ ischemia
Oxidative stress	Reperfusion, leukocyte activation, mitochondrial dysfunction	ROS (O_2_•^−^, H_2_O_2_, •OH), MPO, NOX2	Lipid peroxidation, DNA damage, enzyme inactivation	Cellular injury, impaired vascular reactivity
PI3K/Akt pathway	Cytokines, shear stress, growth factors	PI3K, Akt, mTOR	Cell survival, inflammation modulation	Compensatory response or immune dysregulation
MAPK pathway	Cytokines, ROS, mechanical stress	p38 MAPK, ERK1/2, JNK	Cytokine production, cell activation	Inflammation, apoptosis, organ dysfunction

**Table 3:** Therapeutic approaches targeting CPB-associated inflammation act at multiple levels of the inflammatory cascade, including prevention of blood–surface activation, inhibition of complement and coagulation pathways, suppression of leukocyte and endothelial activation, modulation of intracellular inflammatory signaling, attenuation of oxidative and mitochondrial stress, and extracorporeal removal of circulating inflammatory mediators. Conventional interventions, such as corticosteroids, antifibrinolytic agents, biocompatible CPB circuits, ultrafiltration, and blood-conservation strategies, have been investigated extensively. More recent approaches include cytokine hemoadsorption, targeted complement inhibition, NLRP3 inflammasome blockade, Nrf2 activation, NADPH oxidase inhibition, and modulation of non-coding RNA and epigenetic pathways. Importantly, reduction in inflammatory biomarkers does not necessarily translate into improved clinical outcomes, and several interventions remain investigational or have demonstrated inconsistent results. Current evidence therefore supports a multimodal and individualized approach focused on limiting excessive inflammation while preserving physiological immune, coagulation, and tissue-repair responses. ↓ indicates decreased levels or activity.

Therapeutic Strategy/Intervention	Timing	Principal Molecular/Cellular Target	Proposed Mechanism of Anti-Inflammatory Action	Potential Effects During/After CPB	Current Evidence/Limitations
Corticosteroids (methylprednisolone, dexamethasone)	Pre-CPB/during CPB	NF-κB, cytokine transcription, leukocyte activation	Suppress transcription of pro-inflammatory genes and reduce cytokine, adhesion molecule, and leukocyte activation	↓ IL-6, IL-8, TNF-α; attenuation of leukocyte activation; possible reduction in inflammatory response	Strong biological effect, but large RCTs have not demonstrated routine improvement in major clinical outcomes; hyperglycemia and infection are potential adverse effects.
Aprotinin	Before and during CPB	Serine proteases, kallikrein, plasmin, complement/coagulation interactions	Inhibits proteolytic enzymes and attenuates contact system, fibrinolytic, and inflammatory activation	↓ inflammatory mediator generation; reduced bleeding/transfusion; possible attenuation of CPB-associated inflammation	Effective antifibrinolytic but limited by historical safety concerns; current use is selective.
Tranexamic acid	Preoperative/intraoperative	Plasminogen/plasmin system	Inhibits fibrinolysis and indirectly reduces inflammatory consequences associated with excessive fibrin degradation	Reduced blood loss/transfusion; potential reduction in transfusion-related inflammatory burden	Established blood-conservation therapy; anti-inflammatory effect is mainly indirect.
Epsilon-aminocaproic acid	Intraoperative	Plasminogen/plasmin	Antifibrinolytic activity reduces fibrin degradation and blood loss	↓ transfusion-related inflammatory burden; preservation of hemostasis	Useful antifibrinolytic, but primarily a blood-conservation rather than direct anti-inflammatory strategy.
Statins	Chronic/preoperative; perioperative continuation	NF-κB, endothelial signaling, oxidative stress, NO pathway	Pleiotropic anti-inflammatory, antioxidant, and endothelial-stabilizing effects	Potential ↓ inflammatory biomarkers and endothelial protection	Continue when clinically indicated; initiating solely to prevent CPB inflammation is not supported by large RCTs.
Beta-blockers	Preoperative/perioperative	Adrenergic signaling	Reduction in catecholamine-mediated inflammatory and oxidative responses	Potential reduction in postoperative inflammatory stress and AF	Primarily cardiovascular therapy; not established as dedicated anti-inflammatory CPB therapy.
NSAIDs/COX inhibition	Selected postoperative use	COX-1/COX-2, prostaglandins	Suppresses arachidonic-acid-derived prostaglandin synthesis	Potential reduction in pain and inflammatory signaling	Limited by renal, bleeding, and cardiovascular toxicity; not routine therapy specifically for CPB-SIRS.
Selective COX-2 inhibitors	Postoperative	COX-2/PGE2	Reduces prostaglandin-mediated inflammation	Potential reduction in inflammatory symptoms	Cardiovascular and renal safety concerns limit routine use.
Vitamin C	Pre/intra/postoperative	ROS, oxidative stress, endothelial function	Scavenges ROS and may regenerate endogenous antioxidants	Potential reduction in oxidative injury and endothelial dysfunction	Biological rationale is strong, but clinical evidence remains heterogeneous.
Vitamin E/α-tocopherol	Pre/perioperative	Lipid peroxidation	Limits membrane lipid oxidation	Potential reduction in oxidative injury	Limited clinical evidence in modern CPB.
N-acetylcysteine (NAC)	Pre/intra/postoperative	Glutathione/ROS	Replenishes glutathione and attenuates oxidative stress	Potential ↓ oxidative stress, endothelial injury, and renal injury	Trials have shown inconsistent clinical benefit.
Coenzyme Q10	Pre/perioperative	Mitochondrial electron transport/ROS	Supports mitochondrial function and antioxidant capacity	Potential myocardial and mitochondrial protection	Limited clinical evidence; mainly investigational.
Melatonin	Pre/intra/postoperative	Mitochondrial ROS, Nrf2, apoptosis	Antioxidant, mitochondrial-stabilizing and anti-inflammatory effects	Potential reduction in oxidative stress and organ injury	Promising experimental evidence; insufficient evidence for routine CPB use.
Allopurinol	Pre/intraoperative	Xanthine oxidase	Reduces ROS generation during ischemia–reperfusion	Potential reduction in oxidative myocardial injury	Experimental/limited clinical evidence.
Methylene blue	Intra/postoperative	NO–cGMP pathway	Inhibits excessive NO-mediated vasodilation and may reduce vasoplegic physiology	Improved vascular tone in vasoplegia; indirect control of inflammatory hemodynamic consequences	Used selectively for vasoplegia rather than general SIRS suppression.
Inhaled nitric oxide (iNO)	During/post-CPB	NO/cGMP, pulmonary vascular signaling	Selective pulmonary vasodilation and potential anti-inflammatory effects	↓ pulmonary vascular resistance; possible pulmonary protection	Organ-specific therapy; clinical benefit depends on indication.
Prostacyclin/epoprostenol	Intra/postoperative	cAMP, platelet/endothelial signaling	Vasodilation and inhibition of platelet activation	Potential improvement of microcirculation and pulmonary vascular function	Limited evidence as a systemic anti-inflammatory intervention.
Milrinone	Intra/postoperative	cAMP/PKA	Vasodilation, modulation of leukocyte/platelet function, improved cardiac output	Potential reduction in microcirculatory dysfunction and inflammatory consequences of low output	Primarily hemodynamic therapy; anti-inflammatory effects are secondary.
C1 esterase inhibitor	Pre/intraoperative	Classical/contact complement activation	Suppresses complement and kallikrein activation	Potential ↓ C3a/C5a and leukocyte activation	Mechanistically attractive; clinical evidence remains heterogeneous.
C5 inhibition		C5/C5a/C5b-9	Blocks terminal complement activation	Potential reduction in anaphylatoxins and membrane attack complex formation	Experimental/not routine in CABG.
C5a receptor antagonism		C5a–C5aR1	Prevents C5a-mediated neutrophil and endothelial activation	Potential reduction in leukocyte recruitment and endothelial injury	Experimental.
Heparin-coated CPB circuits	During CPB	Contact activation/complement/coagulation	Improves blood–surface biocompatibility and reduces protein adsorption and cellular activation	Potential ↓ complement, leukocyte activation, and inflammatory mediator release	Important non-pharmacological adjunct; clinical benefit varies.
Biocompatible polymer-coated circuits	During CPB	Blood–surface interaction	Reduces activation caused by artificial surfaces	Potential reduction in complement and inflammatory activation	Useful biocompatibility strategy.
Leukocyte filtration/depletion	During CPB/early postoperative	Activated neutrophils/leukocytes	Removes activated leukocytes and reduces leukocyte-mediated ROS/protease injury	Potential ↓ pulmonary, myocardial and systemic inflammatory injury	Results inconsistent; timing and patient selection remain important.
Hemofiltration/ultrafiltration	During CPB	Cytokines, inflammatory mediators, fluid overload	Removes plasma water and selected inflammatory mediators	Hemoconcentration, fluid management, possible ↓ cytokine burden	Established for fluid management; anti-inflammatory effect is variable.
Modified ultrafiltration (MUF)	Immediately after CPB	Cytokines, fluid overload	Concentrates blood and removes plasma water/inflammatory mediators	Potential ↓ IL-6 and other mediators; improved hemodynamics	More established in pediatric surgery; adult CABG evidence less consistent.
Hemoadsorption/cytokine adsorption	During CPB/early postoperative	IL-6, IL-8, TNF-α and other mediators	Adsorptive removal of circulating inflammatory molecules	↓ circulating cytokine burden; possible reduction in AKI	Recent evidence suggests lower AKI in some analyses but no clear mortality/RRT benefit; routine use remains uncertain.
CytoSorb^®^	Intraoperative/postoperative	Cytokines and middle-molecular-weight mediators	Extracorporeal adsorption	Potential ↓ cytokine levels	Evidence is heterogeneous; randomized trials have not established clear clinical benefit.
oXiris^®^/adsorptive membranes	During CPB/postoperative	Cytokines, endotoxin	Adsorption of inflammatory mediators and endotoxin	Potential reduction in inflammatory burden	Limited cardiac surgery evidence.
Endotoxin adsorption	Intra/postoperative	Circulating endotoxin	Removal of endotoxin and reduction in TLR4 stimulation	Potential ↓ TLR4/NF-κB activation	Mainly investigational in CPB.
Polymyxin-B hemoperfusion	Selected high-risk settings	Endotoxin	Adsorption of endotoxin	Potential reduction in endotoxin-driven inflammation	Evidence mainly from sepsis; limited direct CABG evidence.
TLR4 inhibition		TLR4/MyD88	Blocks DAMP/PAMP-induced innate immune activation	Potential ↓ NF-κB/MAPK activation and cytokine release	Experimental.
MyD88 inhibition		MyD88 adaptor	Interrupts TLR-dependent inflammatory signaling	Potential ↓ NF-κB and MAPK activation	Experimental.
NF-κB inhibition		IKK/NF-κB	Prevents transcription of pro-inflammatory genes	Potential ↓ TNF-α, IL-1β, IL-6, adhesion molecules	Strong mechanistic rationale, but systemic inhibition may impair host defense.
p38 MAPK inhibition		p38 MAPK	Suppresses stress-induced inflammatory transcription	Potential ↓ cytokines and leukocyte activation	Experimental.
JNK inhibition		JNK/AP-1	Reduces stress-induced inflammatory and apoptotic signaling	Potential cellular protection	Experimental.
PI3K/Akt modulation		PI3K/Akt	Modulates cell survival, endothelial function, and immune metabolism	Potential cytoprotection and endothelial stabilization	Highly context-dependent; not routine anti-inflammatory therapy.
mTOR modulation		mTOR/metabolism	Modifies immune-cell metabolic programming and autophagy	Potential reduction in maladaptive immune activation	Experimental.
JAK/STAT inhibition		JAK/STAT	Reduces cytokine-induced transcriptional signaling	Potential reduction in IL-6/STAT3-driven inflammation	Experimental; systemic immunosuppression is a concern.
NLRP3 inflammasome inhibition		NLRP3/ASC/caspase-1	Prevents IL-1β/IL-18 maturation and pyroptosis	Potential reduction in myocardial, renal, and endothelial inflammation	Emerging experimental strategy.
Caspase-1 inhibition		Caspase-1	Blocks inflammasome-mediated cytokine maturation and pyroptosis	Potential ↓ IL-1β/IL-18 release	Experimental.
IL-1 receptor antagonism	Experimental/selected clinical concept	IL-1β/IL-1R	Blocks downstream IL-1 inflammatory signaling	Potential reduction in systemic inflammation	Not established for routine CPB.
IL-6 receptor blockade		IL-6/IL-6R/STAT3	Suppresses IL-6-driven acute-phase and inflammatory signaling	Potential reduction in excessive systemic inflammation	Insufficient evidence for routine CABG.
TNF-α inhibition		TNF/TNFR/NF-κB	Blocks a major inflammatory cytokine	Potential reduction in cytokine amplification	Infection/immunosuppression concerns; not routine.
IL-10 augmentation		Anti-inflammatory cytokine signaling	Suppresses macrophage and cytokine activation	Potential promotion of inflammatory resolution	Experimental.
Nrf2 activation	Experimental/pharmacological	Nrf2/ARE	Increases endogenous antioxidant and cytoprotective genes	↓ ROS, oxidative injury, and inflammatory signaling	Promising molecular target.
NADPH oxidase inhibition		NOX1/NOX2/NOX4	Reduces ROS generation upstream of inflammatory amplification	Potential reduction in oxidative and endothelial injury	Experimental.
Mitochondrial antioxidants (e.g., MitoQ)		Mitochondrial ROS	Selective mitochondrial ROS scavenging	Potential preservation of mitochondrial function and reduction in inflammasome activation	Experimental.
eNOS-supportive therapy/BH4		eNOS coupling	Maintains eNOS NO production and prevents eNOS-derived superoxide	Potential endothelial protection	Experimental.
Remote ischemic preconditioning (RIPC)	Before CPB	RISK/SAFE, mitochondrial and inflammatory pathways	Activates endogenous protective signaling and reduces ischemia–reperfusion injury	Potential myocardial, renal and endothelial protection	Large RCTs have shown inconsistent clinical benefit.
Ischemic postconditioning	During reperfusion	Mitochondrial permeability transition/ROS	Modulates reperfusion-associated oxidative and mitochondrial injury	Potential myocardial protection	Experimental/variable clinical evidence.
Volatile anesthetics	Intraoperative	Mitochondrial KATP/RISK/SAFE signaling	Pharmacological preconditioning and modulation of ischemia–reperfusion responses	Potential myocardial protection and modulation of inflammation	Evidence mixed; not primarily anti-inflammatory.
Propofol	Intraoperative	ROS/mitochondrial signaling	Antioxidant and anti-inflammatory properties	Potential reduction in oxidative stress	Clinical relevance to CPB-SIRS remains uncertain.
Dexmedetomidine	Perioperative	α2-adrenergic and inflammatory pathways	Sympatholysis and possible suppression of cytokine/oxidative responses	Potential ↓ inflammatory biomarkers and postoperative stress	Clinical evidence is heterogeneous.
Ketamine	Perioperative	NMDA receptor/inflammatory signaling	May modulate cytokine and neuroinflammatory responses	Potential reduction in inflammatory response	Primarily anesthetic/analgesic; anti-inflammatory evidence variable.
Tight but safe glycemic control	Intra/postoperative	Hyperglycemia/ROS/NF-κB	Limits glucose-driven oxidative and inflammatory signaling	Potential reduction in infection and organ injury	Avoid severe hyperglycemia; overly intensive control increases hypoglycemia risk.
Goal-directed hemodynamic therapy	During/post-CPB	Microcirculation/endothelial stress	Maintains oxygen delivery and reduces ischemia-driven inflammatory signaling	Potential reduction in organ injury and secondary inflammation	Organ-protective rather than direct anti-inflammatory therapy.
Lung-protective ventilation	During/post-CPB	Pulmonary mechanotransduction	Reduces ventilator-induced inflammatory signaling and alveolar injury	Potential reduction in pulmonary SIRS/ARDS	Important supportive strategy.
Normothermia/controlled rewarming	During CPB	ROS, endothelial and mitochondrial stress	Limits excessive inflammatory and oxidative responses during temperature transitions	Potential reduction in reperfusion injury	Perfusion-management strategy.
Minimization of CPB duration	Intraoperative	Multiple inflammatory pathways	Reduces duration of blood–surface exposure and ischemia	Potential ↓ cytokine and complement activation	Fundamental surgical/perfusion strategy.
Off-pump CABG	Operative strategy	Blood–surface interaction	Avoids exposure to CPB circuit	Reduced activation of complement, leukocytes, and coagulation	May reduce some inflammatory markers; clinical advantages are procedure- and patient-dependent.
Cell salvage/blood-conservation strategies	Intra/postoperative	Transfusion-associated inflammation	Reduce exposure to allogeneic blood products	Potential reduction in inflammatory and infectious complications	Primarily patient blood-management strategy.
Restrictive transfusion strategy	Intra/postoperative	Transfusion-related inflammation	Reduces exposure to transfusion-associated inflammatory mediators	Potential reduction in inflammatory complications	Individualize according to oxygen delivery and clinical context.
Early enteral nutrition	Postoperative	Gut barrier/immune metabolism	Maintains intestinal barrier and may reduce bacterial translocation	Potential reduction in endotoxin/TLR4-driven inflammation	Supportive strategy.
Omega-3 fatty acids	Pre/peri/postoperative	Specialized pro-resolving lipid mediators	Modulates eicosanoid synthesis and inflammatory resolution	Potential reduction in inflammation and AF	Evidence mixed.
Resolvin-based therapies		Resolution pathways	Promote termination of inflammation without generalized immunosuppression	Potential enhancement of inflammatory resolution	Experimental.
MicroRNA-based therapies		miRNA-dependent gene regulation	Modulates NF-κB, MAPK, PI3K/Akt, NLRP3 and endothelial pathways	Potential highly targeted molecular suppression of inflammation	Experimental.
Extracellular-vesicle modulation		Intercellular inflammatory signaling	Modifies transfer of inflammatory miRNAs/proteins	Potential reduction in systemic inflammatory signaling	Experimental.
Epigenetic modulation		DNA methylation, histone modification	Alters inflammatory gene transcription	Potential long-term modulation of inflammatory phenotype	Experimental.
Mesenchymal stromal cell-derived exosomes		miRNA/paracrine signaling	Potential inhibition of NF-κB/NLRP3 and promotion of tissue repair	Potential reduction in inflammation and organ injury	Experimental; not routine CABG.

## Data Availability

No new data were created or analyzed in this study. Data sharing is not applicable to this article.

## Abbreviations

CPB	Cardiopulmonary Bypass
DAMPs	Damage-Associated Molecular Patterns
PAMPs	Pathogen-Associated Molecular Patterns
PRRs	Pattern Recognition Receptors
TLR (TLR2, TLR4)	Toll-Like Receptor
NLRs	NOD-Like Receptors
NLRP3	NACHT, LRR, and PYD domains-containing protein 3
RAGE	Receptor for Advanced Glycation End-products
PAR-1	Protease-Activated Receptor 1
TNF-α	Tumor Necrosis Factor-alpha
IL (IL-1β, IL-6, IL-8, IL-10, IL-12, IL-18, IL-23)	Interleukin
IFNs	Interferons
MCP-1	Monocyte Chemoattractant Protein-1
SIRS	Systemic Inflammatory Response Syndrome
ICAM-1	Intercellular Adhesion Molecule-1
VCAM-1	Vascular Cell Adhesion Molecule-1
vWF	von Willebrand Factor
LPS	Lipopolysaccharide
PSGL-1	P-Selectin Glycoprotein Ligand-1
PLAs	Platelet-Leukocyte Aggregates
CD14/CD16/CD40/CD62P; CD40L	Cluster of Differentiation
CR1/CR3	Complement Receptor 1/3
FcγR	Fc-gamma Receptor
GPVI	Glycoprotein VI
MyD88	Myeloid Differentiation Primary Response 88
TRIF	TIR-domain-containing Adapter-inducing Interferon-β
NF-κB	Nuclear Factor Kappa-light-chain-enhancer of Activated B Cells
IKK	IκB Kinase
IκBα	Inhibitor of Nuclear Factor Kappa B Alpha
IRF3	Interferon Regulatory Factor 3
ASC	Apoptosis-associated Speck-like Protein Containing a CARD
PI3K	Phosphoinositide 3-Kinase
Akt	Protein Kinase B
mTOR	Mammalian Target of Rapamycin
MAPK	Mitogen-Activated Protein Kinase
p38 MAPK	p38 Mitogen-Activated Protein Kinase
ERK1/2	Extracellular Signal-Regulated Kinases 1/2
JNK	c-Jun N-terminal Kinase
JAK/STAT	Janus Kinase/Signal Transducer and Activator of Transcription
TGF-β/SMAD	Transforming Growth Factor-beta/Small Mothers Against Decapentaplegic
Nrf2	Nuclear Factor Erythroid 2-Related Factor 2
ROS	Reactive Oxygen Species
MPO	Myeloperoxidase
NOX2	NADPH Oxidase 2
NETs	Neutrophil Extracellular Traps
NADPH	Nicotinamide Adenine Dinucleotide Phosphate
t-PA	Tissue Plasminogen Activator
TAFI	Thrombin-Activatable Fibrinolysis Inhibitor
ADP	Adenosine Diphosphate
TXA_2_	Thromboxane A2
PF4	Platelet Factor 4
MAC	Membrane Attack Complex (C5b-9)
fMLP	Formyl-Methionyl-Leucyl-Phenylalanine
C3, C5, C3a, C5a, C5b-9	Complement components C3 and C5, anaphylatoxins C3a and C5a, and membrane attack complex C5b-9
RCTs	Randomized Controlled Trials
COX/COX-1/COX-2	Cyclooxygenase/Cyclooxygenase-1/Cyclooxygenase-2
PGE2	Prostaglandin E2
AP-1	Activator Protein 1
PI3K/Akt	Phosphoinositide 3-Kinase/Protein Kinase B
STAT3	Signal Transducer and Activator of Transcription 3
IL-1R	Interleukin-1 Receptor
IL-6R	Interleukin-6 Receptor
TNFR	Tumor Necrosis Factor Receptor
ARE	Antioxidant Response Element
NO	Nitric Oxide
cGMP	Cyclic Guanosine Monophosphate
cAMP	Cyclic Adenosine Monophosphate
PKA	Protein Kinase A
NOX1/NOX2/NOX4	NADPH Oxidase 1/2/4
eNOS	Endothelial Nitric Oxide Synthase
BH4	Tetrahydrobiopterin
MitoQ	Mitochondrial-targeted Coenzyme Q10 (Mitoquinol)
CABG	Coronary Artery Bypass Grafting
AF	Atrial Fibrillation
ARDS	Acute Respiratory Distress Syndrome
AKI	Acute Kidney Injury
RRT	Renal Replacement Therapy
MUF	Modified Ultrafiltration
NMDA	N-methyl-D-aspartate
RISK	Reperfusion Injury Salvage Kinase
SAFE	Survivor Activating Factor Enhancement
KATP	ATP-sensitive Potassium Channel
miRNA/MicroRNA	MicroRNA
DNA	Deoxyribonucleic Acid
